# A hierarchical, count-based model highlights challenges in scATAC-seq data analysis and points to opportunities to extract finer-resolution information

**DOI:** 10.1186/s13059-025-03735-y

**Published:** 2025-09-17

**Authors:** Aaron Wing Cheung Kwok, Heejung Shim, Davis J. McCarthy

**Affiliations:** 1 Bioinformatics and Cellular Genomics, St Vincent’s Institute of Medical Research, 3065 Fitzroy, VIC Australia; 2 Melbourne Integrative Genomics, University of Melbourne, 3010 Parkville, VIC Australia; 3 School of Mathematics and Statistics, Faculty of Science, University of Melbourne, 3010 Parkville, VIC Australia; 4 Faculty of Medicine, Dentistry and Health Sciences, University of Melbourne, 3010 Parkville, VIC Australia

## Abstract

**Background:**

Data from Single-cell Assay for Transposase Accessible Chromatin with Sequencing (scATAC-seq) is highly sparse. While current computational methods feature a range of transformation procedures to extract meaningful information, major challenges remain.

**Results:**

Here, we discuss the major scATAC-seq data analysis challenges such as sequencing depth normalization and region-specific biases. We present a hierarchical count model that is motivated by the data generating process of scATAC-seq data. Our simulations show that current scATAC-seq data, while clearly containing physical single-cell resolution, are too sparse to infer true informational-level single-cell, single-region of chromatin accessibility states.

**Conclusions:**

While the broad utility of scATAC-seq at a cell type level is undeniable, describing it as fully resolving chromatin accessibility at single-cell resolution, particularly at individual locus level, may overstate the level of detail currently achievable. We conclude that chromatin accessibility profiling at true single-cell, single-region resolution is challenging with current data sensitivity, but that it may be achieved with promising developments in optimizing the efficiency of scATAC-seq assays.

**Supplementary Information:**

The online version contains supplementary material available at 10.1186/s13059-025-03735-y.

## Introduction

**Fig. 1:**
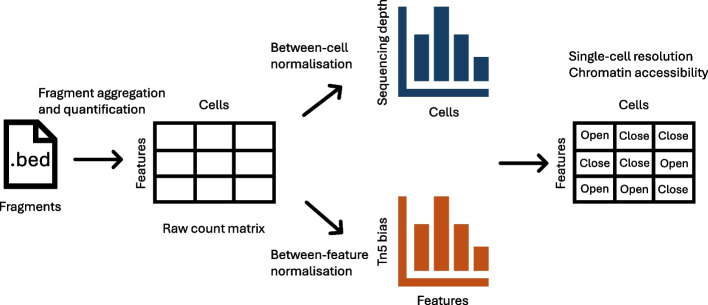
Conceptual diagram for key challenges in typical scATAC-seq data analysis, including fragment aggregation and quantification, between-cell normalization, between-feature normalization, and interpreting chromatin accessibility at single-cell resolution

Single-cell Assay for Transposase Accessible Chromatin with sequencing (scATAC-seq) has established itself as one of the most popular assays for interrogating chromatin accessibility at single-cell resolution [1]. The assay relies on Tn5 transposase which simultaneously fragments accessible DNA regions and integrates adapter sequences, during a process termed “tagmentation” [2]. The DNA fragments from each single cell are then sequenced and quantified which serves as the entry point for data analysis. However, computational analyses of said data are exceptionally challenging due to the data readout of scATAC-seq being sparse, with over 90% of the entries in the count matrix being zeros [3]. This challenge motivates the development of a plethora of novel computational tools to answer meaningful questions about chromatin accessibility. Here, we describe a typical computational workflow for analyzing scATAC-seq data and the major challenges associated with each step (Fig. 1). Starting from the initial readout, i.e., DNA fragments, feature engineering is necessary to group fragments from the whole genome into regions of interest. Using this set of regions of interest, a count matrix can be obtained for various downstream analysis tasks. Next, normalization is typically performed to remove between-cell and/or between-region technical biases, which is usually followed by dimension reduction. Using low-dimensional representations, more concrete biological questions can be addressed, such as cell type annotation, differential accessibility, and motif enrichment.

Although these computational steps are highly analogous to single-cell transcriptome analyses, extreme data sparsity presents unique challenges at each stage of analysis. Below, we elaborate on 4 major challenges in this typical pipeline that remain largely unsolved, with little consensus on the best way to approach them.

## Quantifying chromatin accessibility: concepts and considerations

Like other single-cell modalities, most analysis workflows for scATAC-seq data start with a count matrix. However, quantifying chromatin accessibility is not as straightforward as quantifying gene expression. First, genomic features for scATAC-seq are ambiguous and not standardized, unlike in transcriptomics where features are defined by well-annotated genes and transcripts. In scATAC-seq analyses, researchers will either divide the whole genome into fixed-width windows or identify signal-enriched regions using peak callers to limit the analysis to biologically relevant regions of interest. The choice is usually up to users’ preferences, but occasionally determined by the strategy employed by a specific computational tool (Table 1). In this study, we stick to the fixed-width (500bp) peaks called using the ArchR pipeline [4] where applicable, because with uniform peak lengths the effects of various biases discussed in the following sections can be isolated with one less variable to consider. For the “Major challenge 2: region-specific bias” section, where region-level bias is discussed, we opt to use the original peak set which is of variable lengths, as (1) more precise peak definitions can impact GC-content quantification, and (2) the effects of peak lengths are generally uniform across samples, analogous to the effects of gene lengths in RNA-seq [5].

Second, within the defined features (be it fixed-width windows or called peaks), whether to count individual Tn5 insertion events or the presence of whole fragments is another topic up for debate. As a result, for the same raw fragment file, different counting strategies can generate different count matrices. These intricacies are discussed in great detail by Miao and Kim [6], who propose paired insertion counts (PIC) as the preferred quantification method for scATAC-seq data. Briefly, for a given region, if the pair of insertions of a fragment are both within the region, count as one (pair); if only one insertion is within the region, also count as one (pair). This counting scheme resolves many false positive cases, as long-spanning fragments with both insertion events happening outside the target region will not be counted. The advantage of using PIC is twofold: it has attractive statistical properties for modeling purposes and, as pointed out by Miao and Kim [6] and Martens et al. [7], the quantitative nature of the scATAC-seq readout can be related to biology. As such, here we opt to frame our discussion around PIC quantification of chromatin accessibility from scATAC-seq data.

## Major challenge 1: sequencing depth normalization

Sequencing depth variation between cells is a common source of unwanted variation in any single-cell sequencing data. If not properly accounted for, the variation in sequencing depth can be the largest source of between-cell variation and mask biological heterogeneity. In single-cell RNA-seq (scRNA-seq) data analyses, variation in sequencing depth is usually dealt with via normalization prior to downstream analysis. For scATAC-seq data, the most widely used option is term frequency-inverse document frequency (TF-IDF) normalization. It is implemented with different flavors in popular tools such as Signac [8], ArchR [4], scOpen [3], and Cell Ranger ATAC [9] (summarized in Table 1). Importantly, TF-IDF preserves the region $\times$ cell dimensions of the count matrix without prior aggregation. However, benchmark studies show that it is often ineffective in removing library size effects [10] and, despite its popularity, there is little discussion on why that is the case. As such, choosing a particular TF-IDF flavor is mostly based on heuristics, personal preferences, and default settings in software packages.

**Table 1:** Comparison of different flavors of TF-IDF implementation and the default counting schemes used. Note that the counting schemes are effectively user choices, as one can always manually swap out the counting scheme and/or TF-IDF variant used. Here we only highlight default options provided by the authors

Method	TF-IDF	Features	Counting	Binarise
Signac [8]	$\textrm{log}(\text {TF} \times 10^4 \times \text {IDF} + 1)$	Peaks	Fragments	No
ArchR [4], scOpen [3]	$\text {TF} \times \textrm{log}(\text {IDF}+1)$	500bp bins	Insertions	Yes
Cusanovich [11]	$\text {TF} \times \textrm{log}(\text {IDF}+1)$	5kbp bins	Insertions	Yes
Hill [12]	$\textrm{log}(\text {TF} \times 10^5 + 1) \times \textrm{log}(\text {IDF})$	Peaks/bins	Insertions	Yes
Cell Ranger ATAC [9]	$\textrm{log}(\text {IDF})$	Peaks	Insertions	No

### TF-IDF approaches are counterproductive in removing sequencing depth biases

To explain the observed inefficiency of library size effect correction from TF-IDF based methods [10], we will elaborate on its calculation and theoretical limitations. As the name suggests, TF-IDF is the product of two distinct parts: term frequency (TF) and inverse document frequency (IDF). Here, we unpack the two parts of TF-IDF and identify inherent limitations in its application as a default normalization strategy for sequencing depth variation in scATAC-seq data.

#### Term frequency

We work with an $N \times P$ “count matrix” $\varvec{X}$ which holds information about the number of observed counts in *N* cells and *P* features. The features can represent either peaks or bins depending on the upstream data pre-processing approach. We let $i \in \{1, \dots , N\}$ index cells and $j \in \{1, \dots , P\}$ index features, so that $x_{ij}$ is the observed count of the *j*th feature in the *i*th cell.

The term frequency transformation of a particular count value is defined as the count value divided by the sum of counts over all features in the same cell as the count value,

1 $$\begin{aligned} \text {TF}_{ij} = \frac{x_{ij}}{\sum _{j^{\prime } = 1}^{P}x_{ij^{\prime }}} . \end{aligned}$$

We can compare this value to counts per ten thousand (CPTT) commonly used in scaling scRNA-seq counts:

2 $$\begin{aligned} \text {CPTT}_{ij} = \frac{x_{ij}}{\sum _{j^{\prime } = 1}^{P}x_{ij^{\prime }}} \times 10^4 . \end{aligned}$$

Clearly, these two quantities are identical except for the scaling factor of $10^4$. In bulk RNA-seq terminology, it is equivalent to counts per million (CPM) divided by 100. The smaller scaling factor here is used to account for the overall smaller library sizes observed in single cell assays compared to bulk.

**Fig. 2:**
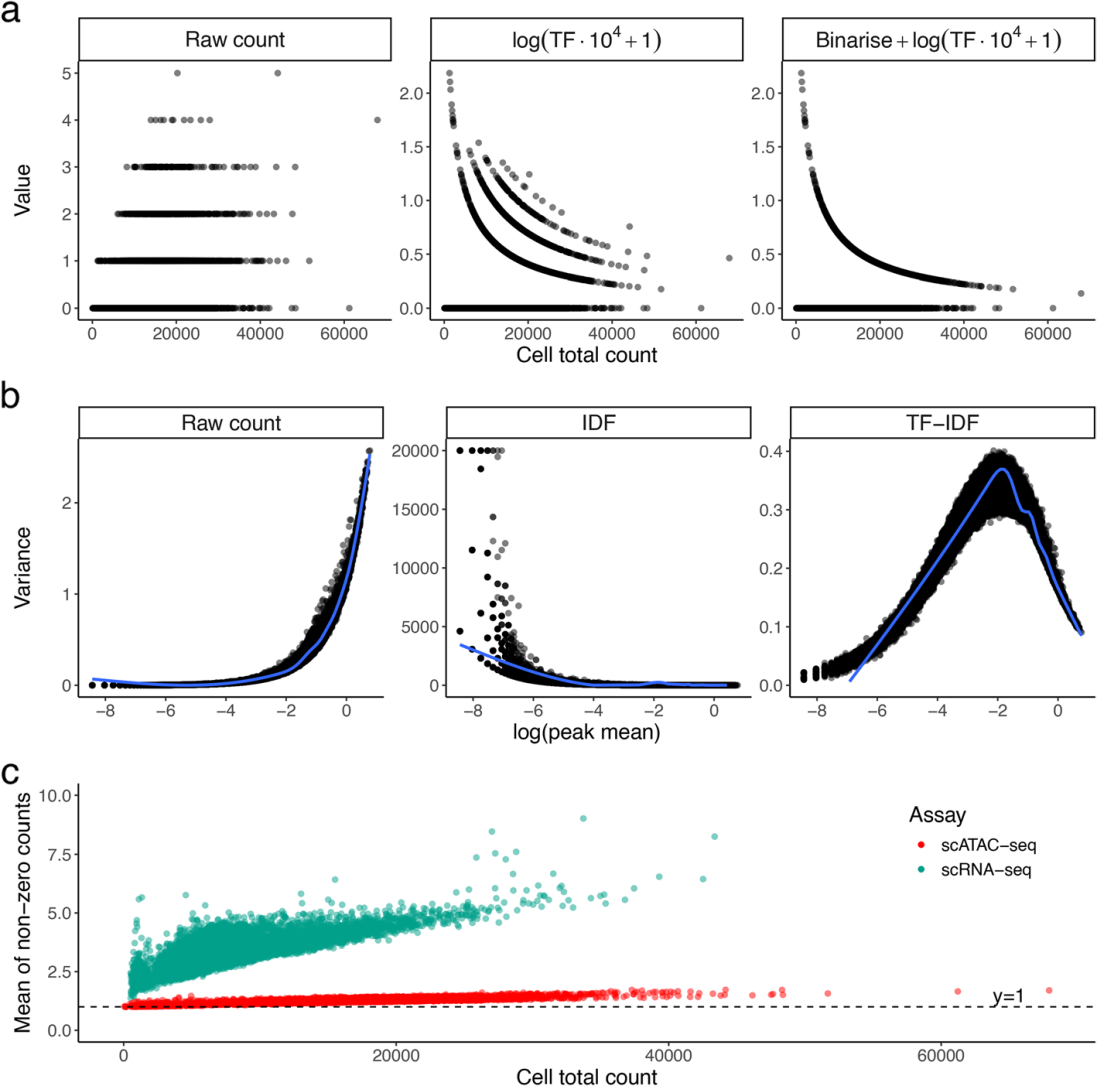
**a** Raw counts and their TF-transformed values for a random region in PBMC10k scATAC-seq dataset, plotted against the total count of each cell. Each dot is a cell. Here the region chr1:1273633-1274133 was chosen for demonstration. **b** Variance of raw count and IDF-transformed values plotted against mean of raw count of each region. Each dot is a region. **c** Mean of non-zero counts in each cell plotted against the total count for both scRNA-seq and scATAC-seq data from the PBMC10k dataset

Dividing by total count is a sound strategy for bulk sequencing as the read counts are often in the magnitudes of hundreds to thousands, with total counts per sample in the millions. However, in scATAC-seq data, most data entries share the same value at either 0 or 1 (comprising of 90–95% of the data), but the total count of each cell is different. Therefore, after TF transformation, the largest variation between cells will naturally be due to their denominators, that is, the total counts per cell or sequencing depth (Fig. 2a). This effect is further exacerbated by binarising the counts before transformation (as done in some popular analyses software, e.g., ArchR, scOpen), which forces all non-zero entries to share the same value of 1 (Fig. 2a). Ironically, the aim of this strategy is to remove sequencing depth variation, but it ends up introducing extra information about library sizes instead.

Due to the large number of genomic regions and likely small number of Tn5 cuts in each region, the majority of observed counts of scATAC-seq data is exactly zero (Additional file 1: Fig. S1). Thus, an increasing sequencing depth will more likely turn a 0 into 1 instead of turning a 1 to a value larger than 1. We observed that the mean of non-zero counts in scATAC-seq rarely goes above 1.2 even in cells with high total counts, which is on average 62.8% lower than that of scRNA-seq data (Fig. 2c). In other words, sequencing depth difference is mostly represented by sparsity and normalization methods that target non-zero values (e.g., dividing by total count or a linear size factor) will not address the problem effectively. This has been a known issue for scRNA-seq, where bulk-based methods like $\textrm{log}(\text {CPM}+1)$ were found to be sub-optimal as they fail to account for exact zeros and the arbitrary choice of pseudocount can introduce subtle bias to the data [13]. TF transformation, being a rehash of CPM, suffers from the same issues as its scRNA-seq counterpart as we observe parallels in count characteristics.

#### Inverse document frequency

IDF is a feature-wise metric that weights features according to their rarity among all features, given by:

3 $$\begin{aligned} \text {IDF}_{j} = \frac{N}{\sum _{i^{\prime } = 1}^{N}x_{i^{\prime }j}} . \end{aligned}$$

We can also rewrite IDF in terms of region mean count $\mu _j$:

4 $$\begin{aligned} \text {IDF}_{j} = \frac{1}{\mu _{j}} . \end{aligned}$$

The intuition behind IDF is to give more weight to regions that are rarely open as they are more likely to correlate with cell-type-specific functions, while less weight is given to regions that are open in most cells as they are likely to be involved in housekeeping functions that are not relevant to cell type. In a normal cell clustering task, this weighting scheme is sensible, but it should not be viewed as a typical “normalization” technique that can transfer to other tasks. Region-wise scaling with the region’s mean $\mu _j$ strengthens the mean-variance relationship across regions (Fig. 2b). To be specific, the variance will be scaled by a factor of $1/\mu _j^2$. Caution has to be exercised when applying IDF transformed counts to models that assume uniform variance as IDF will inherently tend to exacerbate heteroskedasticity in scATAC-seq data.

### What now and what’s next?

To that end, we recommend against treating TF-IDF as a depth normalization method due to the theoretical limitations shown. While we do not deny its utility in tasks such as cell type clustering, the resulting counts are not “depth-normalized.” In many cases, the sequencing depth effect is even exaggerated after TF-IDF transformation, leading to yet another bandage solution in analyses, specifically, removing the first principal component manually before clustering. It is worth noting that the aforementioned statistical pitfalls of TF-IDF might not matter much in dimension reduction tasks as ad-hoc solutions seem to work well. However, using TF-IDF values for more sensitive tasks such as differential accessibility (DA) testing as implemented in Signac [8] can have unknown implications. As pointed out in a benchmark by Teo et al. [14], performing DA analysis with TF-IDF normalization improves concordance with matching bulk data but also increases false discoveries, but the exact reason was not discussed.

Single-cell transcriptomic data analyses have multiple available methods for depth normalization. In contrast, apart from TF-IDF, there is a lack of methods for scATAC-seq data analyses that simply return “depth-normalized counts” with the same dimensions. However, there are tools not based on TF-IDF that incorporate sequencing depth information into downstream tasks without explicitly normalizing with total count or size factor. Instead, they try to learn the relationship between sequencing depth and observed count directly from the data. For example, PeakVI [15] trains a neural net specifically on learning the cell-specific scaling factor; PACS [16] parameterizes the sequencing depth effect as an observation probability which is learnt from count data directly. A recent benchmark [17] also showed that linear regression-based normalization implemented in SnapATAC [18] is more robust for difficult clustering tasks.

## Major challenge 2: region-specific bias

Detection of open chromatin with ATAC-seq heavily relies on the tagmentation activity of the Tn5 enzyme, which has a preference for some genomic sequence characteristics over others, leading to technical variation between regions that does not necessarily reflect differences in local accessibility [19]. For bulk ATAC-seq, strategies have been developed to mitigate the effect of Tn5 cleavage bias on downstream analysis, such as weight matrix scaling (ATACorrect [20]), position dependency models (HINT-ATAC [21]), and *k*-mer based methods like SELMA [22]. Apart from sequence composition, it has been shown that epigenetic features such as DNA motif, shape, and methylation can drive Tn5 preferences [23]. The overall mechanism of Tn5 bias is complex and difficult to quantify accurately. Therefore, to reduce the scope of this study, we chose to showcase GC-content as a representative for region-specific bias, which is a well known factor that drives sample-specific technical bias in DNA sequencing (DNA-seq), Chromatin Immunoprecipitation sequencing (ChIP-seq), and RNA sequencing (RNA-seq) data [24]. For bulk ATAC-seq, normalization with regard to GC-content is also crucial to avoid confounding downstream analysis [5]. Although the same effects should be expected in scATAC-seq as well, there is rarely a bespoke step in pipelines to normalize for GC effects, unless when some aggregation has been done beforehand that amplifies technical bias, e.g., chromVAR aggregating peaks based on motifs [25].

### GC correction methods designed for bulk ATAC-seq mitigate single-cell bias at the pseudobulk level

**Fig. 3:**
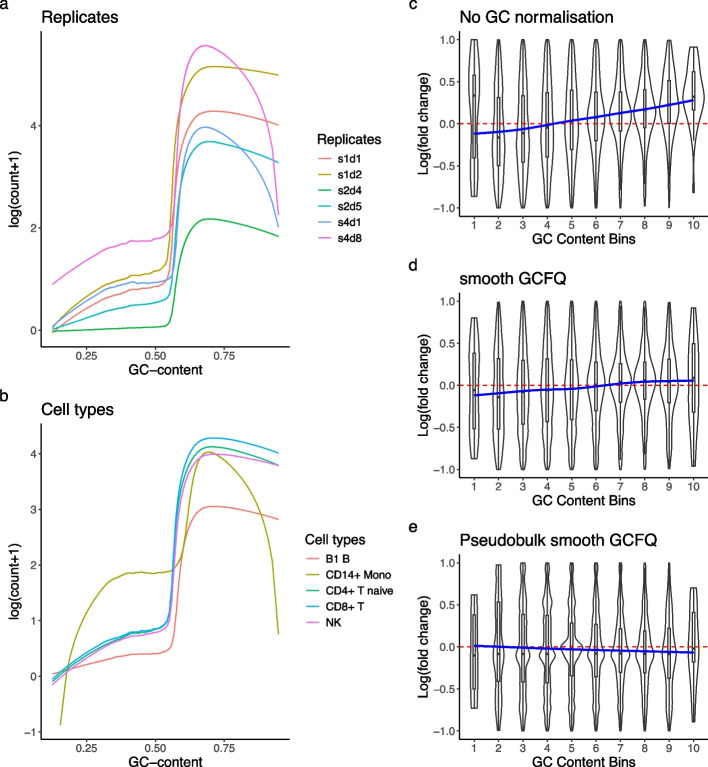
Fitted lowess curves of $\textrm{log}(\text {count+1})$ as a function of GC-content for: **a** 6 of the replicates (s: sequencing batch, d: donor) of CD8+ T cells and **b** 5 of the annotated cell types from donor s1d1 in the Luecken dataset. **c**–**e** Mock null comparison between CD16+ Monocytes. Peaks are sorted into 10 bins according to their GC-content, and log-fold changes (LFC) between the mock groups are plotted against their respective bins. In a null setting, the LFC should be centered at zero. The blue curve represents a generalized additive model (GAM) fit

The effect of GC-content on bulk ATAC-seq readout is well characterized [5], and we observed the same effect on scATAC-seq data (Fig. 3a, b), where regions with higher GC-content tend to have higher mean counts, with the effect varying between replicates of the same cell type and between cell types as seen from the different slopes and shapes of the fitted lowess curves. While such a relationship can be explained by biology due to many accessible regions being gene promoters which often have high GC-content [26], technical variation between regions makes features hard to compare and possibly confound analyses that involve region-to-region comparison. GC bias is also more comparable between replicates within the same sequencing batch than between batches (Fig. 3a), indicating the bias is more of a technical artifact than biology.

Intuitively, it might seem that GC-content would not affect differential accessibility (DA) analyses since comparisons are made across cells for the same genomic region with fixed GC-content. However, because the extent of GC-related biases can vary between libraries, they can still influence the log-fold changes (LFCs) used to assess differences in accessibility [5, 27]. Following Van den Berge et al. [5] and Teo et al. [14], we conducted a mock null DA testing between replicates using an scATAC-seq dataset with 13 donors under identical experimental conditions [28] (Methods “Estimating observation probability$p_{i}$” section). For each annotated cell type, we randomly assigned “control” and “treatment” labels to the replicates to split them into 2 groups. We found that the log-fold change of a region has a bias with respect to its GC-content, instead of being centered at zero as one would predict theoretically for data with minimal biological variation (Fig. 3c, Additional file 1: Fig. S2), consistent with what Van den Berge et al. [5] observed in bulk ATAC-seq data. We repeated the experiment with 20 random splits of donors and found that the bias is persistent (Additional file 1: Fig. S3).

Unfortunately, GC-aware normalization methods for bulk ATAC-seq have limited utility on single-cell level scATAC-seq data. We repeated the above experiment with smooth GC-FQ [5, 29] (“Normalization methods” section) applied to both single-cell level counts and pseudobulked counts. We found that smooth GC-FQ, which performed well on bulk ATAC-seq data, cannot fully remove the effect of GC-content on log-fold change in single-cell level counts (Fig. 3d). However, smooth GC-FQ is more effective on a pseudobulked version of the same data (Fig. 3e). This observation is consistent with its good performance on bulk ATAC-seq data [5], as the pseudobulked counts more closely resemble bulk ATAC-seq counts than does single-cell data.

### What now and what’s next?

Our observations confirm that GC-content bias persists in scATAC-seq data and can systematically skew downstream analyses such as differential accessibility (DA) analysis. Despite previous success in bulk ATAC-seq, GC-aware normalization techniques such as smooth GC-FQ fail to fully correct for this bias at the single-cell level. This issue arises mostly because of the incompatibility between the quantile matching basis of most bulk adjustment methods and the fact that quantiles of a highly sparse scATAC-seq count matrix are predominantly exact zeroes.

Considering the sparseness of scATAC-seq count data, it might be unreasonable to expect a global adjustment method to be effective, as most of the time the only available operations are turning a 0 into a 1 or vice versa. Therefore, a more practical approach is to incorporate region-level bias as a covariate within the analysis task. This approach is commonly used for the most downstream tasks such as computing motif deviation [4], peak-to-gene linkage [8], and analysis of copy number variations [30, 31], but we recommend region-level biases to be considered for common tasks like DA analysis as well. For scRNA-seq, it has been shown that batch effect impacts different genes in different ways, resulting in gene-level batch effects [32], leading us to believe that there is value in considering region-level technical bias in scATAC-seq data as well, instead of generalizing the effect to a single batch covariate that is applied to all features.

## Major challenge 3: interpretation of chromatin accessibility at a single-cell, single-region level

Despite being the main motivation for scATAC-seq, the interpretation of “profiling chromatin accessibility at single-cell resolution” is unclear. A longstanding notion treats chromatin accessibility as binary: a region is either open or closed in a cell. In reality, with two copies of each chromosome in a cell (for autosomes in a diploid organism), the “true” chromatin accessibility state is at least ternary: both chromosomes open, both closed, or one closed and one open. Moreover, recent studies showed that scATAC-seq counts have quantitative information instead [6, 7], as biological factors such as nucleosome turnover rate can contribute to the quantitative observation of chromatin accessibility [7]. To take it even further, it has recently been argued that it is unclear whether euchromatin should even be considered “open” *per se* [33]. As such, depending on the biological assumptions, the interpretation of “chromatin accessibility at single-cell resolution” can vary and thus introduces ambiguity when interrogating scATAC-seq data. These nuances are rarely addressed as most computational analyses are limited to cluster or cell type level, where counts are aggregated and treated as if they were continuous, like gene expression.

What does it mean to truly realize the “single-cell” in scATAC-seq? We interpret this goal to be the ability to tell if each cell is “accessible” at each individual region, following the mainstream assumption of chromatin accessibility being binary. However, as discussed, chromatin biology is highly complex and might not be strictly binary. Therefore to reduce the scope of the study, we aim first to interrogate the simplest case, which is to assume chromatin accessibility is binary at a single cell-single region level, i.e., a cell is either open or closed for a single region.

### A hierarchical model to infer single-cell, single-region chromatin states

With the intention of dealing with all the technical biases listed above and also inferring per-cell, per-region open/closed information from scATAC-seq data, we constructed the following hierarchical model. We work with an $N \times P$ scATAC-seq paired insertion count (PIC) matrix [6]. Let $i=\{1,\dots ,N\}$ index cells and $j=\{1,\dots ,P\}$ index chromatin regions. We define a mixture model that describes the observed count $x_{ij}$ with the following hierarchical structure:

5 $$\begin{aligned} x_{ij} \sim \text {Binomial}(y_{ij}, p_i) , \end{aligned}$$

6 $$\begin{aligned} y_{ij} \sim \left\{ \begin{array}{ll} \text {Poisson}\left(\lambda ^c_{j}s_j\right) & \text {if}\ Z_{ij} = 1 \\ \text {Poisson}\left(\lambda ^c_{j}\right) & \text {if}\ Z_{ij} = 0 \\ \end{array}\right. , \end{aligned}$$

7 $$\begin{aligned} Z_{ij} \sim \text {Bernoulli}(\pi _j) . \end{aligned}$$

Where:

$p_i$ denotes cell-specific observation probability;

$y_{ij}$ denotes true number of paired Tn5 cuts (latent);

$\lambda ^c_{j}$ denotes count rate for closed cells (background count rate due to GC effect);

$s_j$ denotes signal-to-noise ratio;

$\pi _j$ denotes proportion of open cells for a given region.

The motivation for this model specification is to describe biological and technical processes with explainable variables. We have aimed to keep the model as simple, and thus as interpretable, as possible while capturing the most important aspects of the data generation process. We stick to the notion that for a given region, single cells can either be open or closed, as discussed in the “Major challenge 3: interpretation of chromatin accessibility at a single-cell, single-region level” section. The proportion of open cells is denoted by $\pi _j$. In an scATAC-seq experiment, DNA regions are fragmented depending on their accessibility state ($Z_{ij}$), affinity for Tn5 ($\lambda ^c_{j}$), and signal-to-noise ratio ($s_{j}$), but not every accessible region in every cell can be fragmented by Tn5. This property is represented by the Poisson distribution. Lastly, the resulting latent fragments are subjected to technical sampling bias that varies among cells, which is represented by the binomial distribution.

Our model addresses the previously stated major challenges as follows:

- Modeling counts instead of binarised data to extract more information, as suggested by Miao and Kim [6] and Martens et al. [7]. This approach is not inherently contradictory to the assumption of chromatin accessibility being a binary trait. Intuitively, a higher fragment count should indicate a higher confidence of the cell being “open” in a region and vice versa.
- Our modeling approach has the advantage of retaining the region $\times$ cell dimension of the count matrix and requires no arbitrary transformation or prior clustering and cell type annotation.
- Inferring binary state of each cell (open/closed) through using the posterior probability of $Z_{ij}$, i.e., $P(Z_{ij} = 1|\text {data})$.
- Instead of using total count as a scaling factor, using the binomial observation probability $p_i$ is a more faithful representation of fragment dropout. This approach is conceptually similar to the observation probability in the PIC model (Methods “Estimating observation probability$p_i$”) [6].
- Specifying a background rate $\lambda ^c_j$ to be region-specific accounts for region-specific biases such as GC-content variation. In theory, one can further specify $\lambda ^c_j$ to be a function of any known technical effect. In our analysis we chose GC-content to be the representative region-specific effect. Another common region-specific bias can be peak length, which can also be specified as a parameter in the generalized additive model (Methods “Estimating background rate$\lambda ^c_j$”); however, in this study, we chose to demonstrate our findings in a fixed-width peaks setting to simplify our discussion on single-cell accessibility state inference without having to introduce an additional layer of complexity.

We will apply this simple model to address the key challenges in scATAC-seq data outlined above and draw conclusions about current approaches to modeling and analyzing scATAC-seq data.

### Current scATAC-seq data does not have enough information to infer single-cell, single-region level accessibility state

The lack of ground truth makes it difficult to properly evaluate our model on real datasets. Therefore, we first simulated data with a wide range of parameters to the following: (1) quantify the level of information needed to perform accurate inference, and (2) get a rough idea of how real data would behave.

**Fig. 4:**
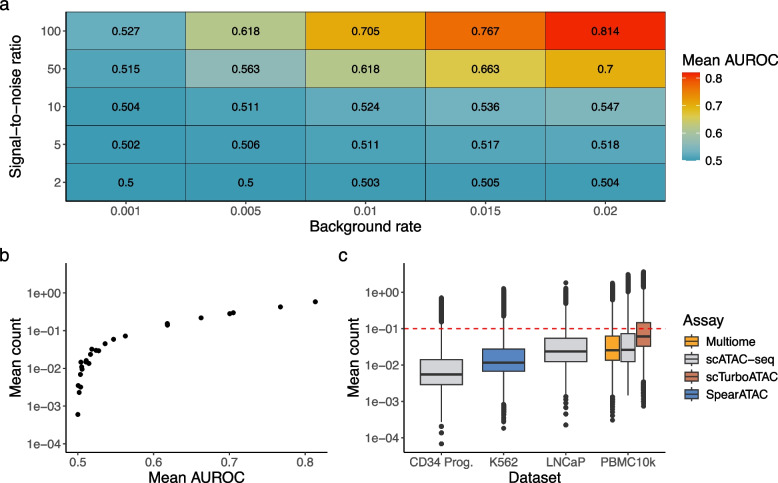
**a** Simulation data with different combinations of background rates and signal-to-noise ratio. $\pi _j$ is fixed to 0.3 for demonstration purposes. For each scenario, simulation is repeated for 30 times and the mean AUROC is calculated. **b** Mean AUROC against mean count of simulated counts. **c** Box plot of peak mean from 6 datasets with varying biology and assays. Red dotted line marks the point where mean count $\ge$ 0.1, corresponding to AUROC $\ge$ 0.55 in our simulations

We simulated 10,000 cells from our hierarchical model with varying background rates $\lambda ^c_j$ and signal-to-noise ratios $s_j$ (Methods “Simulation”). We estimated $p_i$ from data (Methods “Estimating observation probability$p_i$”) and fixed $\pi _j$ to 0.3 for demonstration purposes. Our findings are mostly invariant to the choice of $\pi _j$ (Additional File 1: Fig. S4). For each simulated scenario, $P(Z_{ij} = 1 | x_{ij})$ is calculated using ground truth parameters (Methods “Computation of posterior probability”). The posterior is then evaluated against the ground truth accessibility state of each observation. Each scenario was repeated 30 times and evaluated by the mean area under receiver operating characteristic curve (AUROC) (Fig. 4a). Given that the counts were simulated from the same model and ground truth parameters were used to compute the posterior, we would expect the posterior to be highly informative for identifying cells that are “open,” i.e., high AUROC. The opposite case would suggest that there is a “component collapse” problem, i.e., open cells and closed cells do not have a significant difference in counts and cannot be told apart.

Even with perfect retrieval of parameters, single-cell single-region chromatin states are almost unidentifiable in situations with low $\lambda ^c_j$ or low $s_j$ (Fig. 4a), indicating a severe lack of information in these simulated scenarios. The best case scenario is when both parameters are high ($\lambda ^c_{j} = 0.02, s_j=100$), with mean AUROC 0.84. It is counterintuitive that a low $\lambda ^c_j$ is a more difficult case, as good quality data should have low background noise. However, in the context of the model, the impact of increasing $\lambda ^c_j$ is substantially larger on open cells rather than on the closed cells due to the exponential nature of the Poisson probability mass function, i.e., the ratio of probabilities of sampling a 0 from closed cells versus open cells ($(P(X=0|Z_{ij}=0)/P(X=0|Z_{ij}=1)$) increases exponentially with $\lambda ^c_j$ (Additional file 1: Fig. S5). For low background situations, the ratio is close to 1, implying the chance of sampling 0 count from a closed cell is just as likely as an open cell. Therefore a much larger sample size is needed to distinguish where the 0 is from (i.e., more difficult). With a higher $\lambda ^c_j$, the chance of drawing a 0 from an open cell quickly diminishes, so a 0 count observation is highly likely to be from a closed cell. This imbalance in impact is also consistent with the empirical observation in ChIP-seq that GC bias primarily affects the amplification of open chromatin signals rather than closed peaks [34].

We also found that classification performance correlates strongly with mean count (Fig. 4b), i.e., it is in general easier to correctly identify accessibility states of single cells in peaks with higher counts, which is intuitive as the mean is a function of $\lambda ^c_j$ and $s_j$. This result can serve for practical guidance as one cannot directly observe the underlying parameters in real data. When comparing the mean count of real data against that of simulated counts, we found that in most datasets, less than 25% of peaks have sufficient counts to resemble simulation scenarios with mean AUROC 0.55 or higher (Fig. 4c), indicating more than 75% of features likely have insufficient information to infer chromatin states in single cells. However, scTurboATAC [35], an scATAC-seq assay optimized for Tn5 sensitivity, generated more fragments than other datasets, with 34% of peaks having mean count higher than 0.1 which corresponds to mean AUROC $\ge$ 0.55 in our simulations (Fig. 4c).

### Aggregation and dimensionality reduction can serve as intermediate solutions

As a workaround for the lack of informational resolution in scATAC-seq data, aggregation is necessary to extract useful insights. A common practice in scATAC-seq data analysis is to aggregate information across features via dimension reduction, which is pivotal to most analysis pipelines. For example, Latent Semantic Indexing (LSI) as implemented in Signac [8] and ArchR [4] features TF-IDF transformation followed by Singular Value Decomposition (SVD). This process uses information from all features to produce orthogonal axes that explain the most variance, effectively aggregating features to generate a sensible low dimensional representation of the data. Readers who are familiar with scRNA-seq analysis should not be confused by the new terminologies, as we previously showed that TF-IDF is akin to CPM, and SVD is a generalization of principal component analysis (PCA); so LSI can be understood as library size normalization followed by PCA, which by now is a standard analysis step for scRNA-seq data.

Here we demonstrate an alternative way to utilize aggregated features using our single-cell single-region model without introducing arbitrary normalization steps. Using the 10X multiome PBMC10k dataset, we first naively summed up peak counts based on their nearest genes, reducing the count matrix dimensions from peak $\times$ cell to gene $\times$ cell. Assuming all peaks that share the same nearest gene are correlated in their openness, our proposed model can be applied to the summed counts to derive an estimate of openness for each gene. Then, the model posterior can be directly decomposed with principal component analysis (PCA). We then evaluate the resulting PCs using orthogonal cell type annotation from transcriptome data derived from the same cells.

**Fig. 5:**
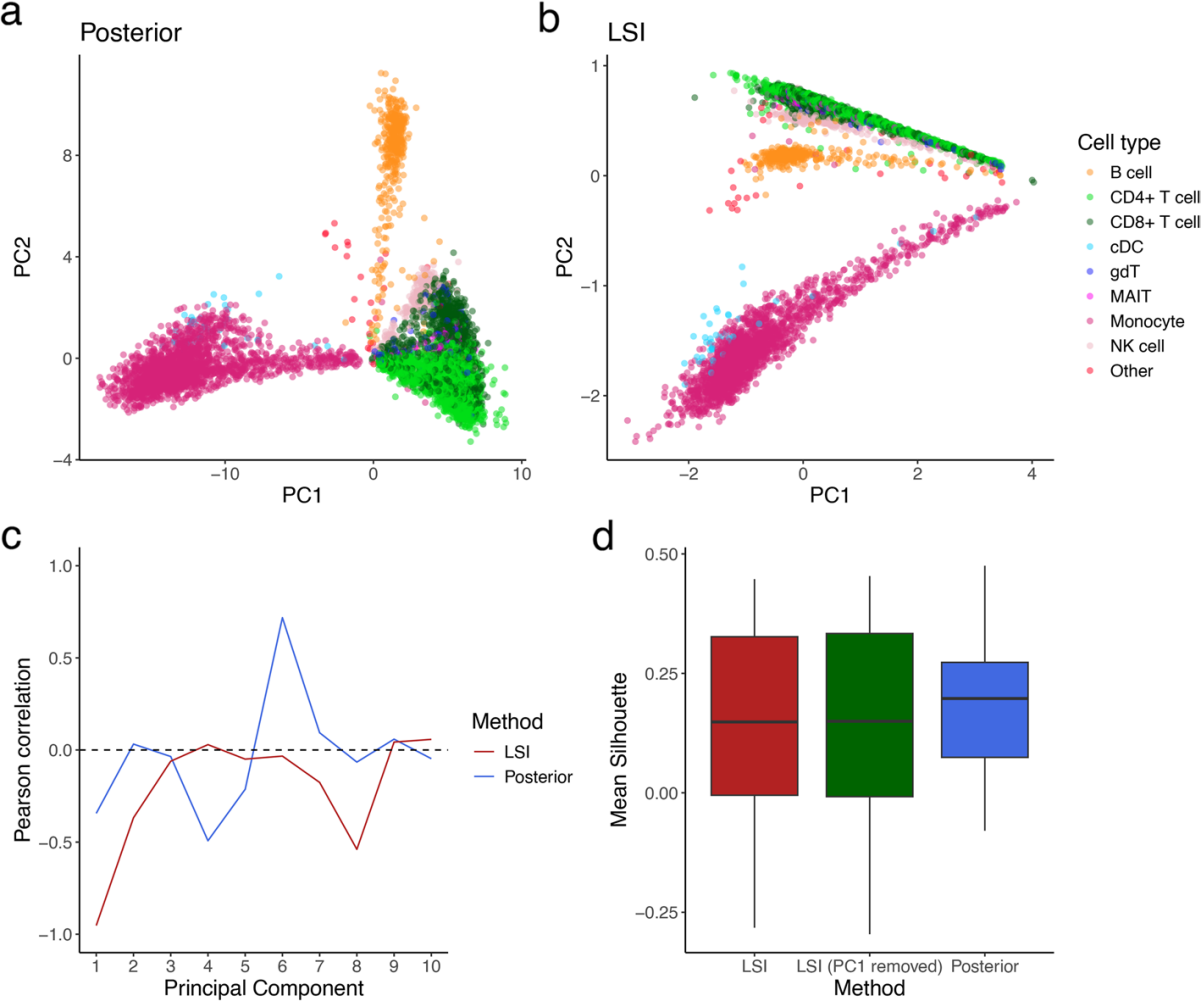
Analysis with 10X multiome PBMC10k dataset. **a–b** The first 2 principal components (PCs) derived from model posterior and LSI respectively. Each dot is a cell, colored by cell type annotation derived from the transcriptome of the same cells. **c** Pearson correlation of the first 10 PCs with library size. **d** Mean silhouette widths for each cell type ($n=8$) derived from the first 30 PCs derived from LSI processed data, LSI with first PC removed, and model posterior

With this approach, we found that PCs obtained from the model can capture biological variation without sequencing depth variation dominating the data (Fig. 5a, b). In particular, the first PC of the model posterior has significantly lower pearson correlation with library size (− 0.34) in contrast with the first PC from TF-IDF normalized data (− 0.95; Fig. 5c). Both approaches exhibit a later PC that correlates strongly with library size (PC8 − 0.54 for LSI and PC4 0.72 for the model posterior). However, these components explain only a small proportion of the variance (0.5% for LSI and 0.2% for the model posterior, Additional file 1: Fig. S9). Given that both methods yield similar results, we argue that the remaining correlation is inconsequential to downstream clustering tasks. It is also not trivial to completely remove via “normalization” as it is likely to be confounded by biological variations, that is, the number of open chromatin regions in a cell (biological) might be indirectly reflected in the total count of the cell (technical).

In addition, we showed that directly using the PCs derived from the model posterior can identify major cell types (Fig. 5a, Additional File 1: Fig. S6–8) and achieve better silhouette scores than LSI, without the need to remove the first PC as an ad-hoc solution (Fig. 5d). Although it is still difficult to definitively say which method is better without performing extensive benchmarks, we showed that even with suboptimal feature aggregation, the modeling approach is at least as good as conventional LSI in terms of identifying major cell types while dealing with library size effects in a statistically sound manner. We observed that removing the first LSI component as recommended in commonly-used workflows resulted in lower silhouette width scores than the model posterior. Thus, this modeling approach, or extensions of it, might be useful for cases where library size and biological variation are strongly related, such that naively removing PCs might risk removing biology.

It must be noted that the demonstrated feature aggregation strategy involves a strong assumption, namely that the proportion of open cells is completely correlated between peaks that are near the same gene. In reality, this is not true and there are more sophisticated ways to aggregate features. For example, chromVAR [25] groups peaks by the presence of certain motifs, effectively reducing the sparse peak $\times$ cell matrix into a smaller motif $\times$ cell matrix. Whereas BROCKMAN [36] summarizes peaks based on *k*-mer frequencies around the insertion sites and Cicero [37] summarizes peaks at the gene level by calculating gene activity scores. Here we merely demonstrate the potential of an explicit statistical inference model to interpret chromatin accessibility while addressing technical artifacts, given that sufficient information is present in the feature.

### What now and what’s next?

We presented a hierarchical count model that is motivated by the data generating process of scATAC-seq data. However, we showed with various simulations that current scATAC-seq data is too sparse to infer true single-cell single-region chromatin accessibility states. While this result might be due to limitations in our assumptions about chromatin accessibility, we reason that if scATAC-seq does not have enough information to recover the simplest binary case, then it is highly likely that more complicated biological models (e.g., ternary, quantitative chromatin states) are also unrecoverable. As such, while the broad utility of scATAC-seq at a cell type level is undeniable, describing it as fully resolving chromatin accessibility at single-cell resolution, particularly at individual locus level, may overstate the level of detail currently achievable. This statement about state inference is not to be confused with the “physical” resolution, as scATAC-seq data is clearly derived from single cells; but the “informational” resolution is not there yet due to data sparsity. It is true that feature aggregation, through dimension reduction is fundamental to single-cell analysis, including gene expression data. Yet these approaches inherently trade granular resolution at individual loci for improved robustness and interpretability at the cell or region-set level. While this represents an effective solution given current data limitations, it also limits our capacity to ask more interesting biological questions. For example, in gene expression data, it is now possible to leverage single-cell level data instead of relying on pseudobulking to answer more complex biological questions, e.g., through context-specific expression QTL mapping [38].

Apart from aggregating features, another approach we have not analyzed is to increase signal by aggregating biologically similar cells prior to analysis. The traditional way is to pseudo-bulk cells of the same cell type and aggregate by either sum or mean, but the concept of “metacells” [39] as a finer grain version of cell type clusters should also be considered. However, the concept of open or closed in aggregated scATAC-seq data is ambiguous as the resolution is no longer single cell and a cell aggregate can contain an arbitrary number of open cells. In this case, a model that treats chromatin accessibility as a quantitative trait, such as the PACS model [16], might be more suitable. Another concern with cell-type or metacell aggregation would be its dependence on low dimension embeddings. Many cell clustering algorithms, including meta-cell methods like SEAcell [40], rely on the constructing a *k*-nearest neighbor (KNN) graph from low dimension embeddings, which in turn relies on proper data preprocessing and normalization. How best to preprocess and normalize are still open questions for scATAC-seq data where the most recommended LSI method has statistical pitfalls and the prevalent assumption of binary-ness is challenged [6, 7]. Similar to feature aggregation, perhaps a softer form of aggregation instead of hard assignment to groups can be considered to boost signals in individual cells.

No matter how sophisticated computational methods get, ultimately the chromatin accessibility information that can be extracted from scATAC-seq is limited by the efficiency of Tn5 transposase insertion [35]. Our previous simulations show that it is possible to reliably infer cell chromatin states within a peak given a sufficient amount of information. However, this can only be achieved by improving the sensitivity of Tn5 transposase itself such that more insertion events can happen. One such example is scTurboATAC [35], in which the sensitivity and versatility of Tn5 transposase were enhanced with optimized experimental workflows. Though this does not guarantee a significant increase in single-cell level information, we believe an experimental approach to address the enormous sparsity in scATAC-seq data is a step to the right direction. Future assay improvements should strive to not only increase signal but also minimize noise to optimize for a better signal-to-noise ratio.

## Conclusion

To conclude, we have provided a general overview of problems in scATAC-seq data analysis, such as fragment quantification, normalization, and interpretation of “chromatin accessibility.” In particular, we showed that the widely used TF-IDF normalization has statistical pitfalls that exacerbate technical bias. We proposed a hierarchical model to infer single-cell chromatin states from scATAC-seq counts. However, our simulations showed that with the sparsity in current scATAC-seq data, it is almost impossible to accurately identify whether a cell is open or closed in a chromatin region. While aggregation and dimensionality reduction allow scATAC-seq data to yield meaningful biological insights at the cell-type level, reliably inferring chromatin accessibility at the resolution of individual loci within individual cells remains a significant challenge with current technologies. To realize this goal, improving the sensitivity of scATAC-seq assays appears to be a promising avenue.

## Methods

### Datasets and preprocessing

#### Downloading data

All datasets used in this study are publicly available (Table 2). The PBMC10k datasets (scATAC-seq, scRNA-seq, and Multiome) were downloaded from the 10X Genomics website. (Link to scATAC-seq, scRNA-seq, Multiome).

**Table 2:** Summary of datasets used

Dataset	Assay	Citation	Accession	# cells	# features
PBMC10k	scATAC-seq	10X Genomics [41]	10X website	10,246	191,833
PBMC10k	scRNA-seq	10X Genomics [42]	10X website	11,922	22,302
PBMC10k	Multiome	10X Genomics [43]	10X website	9829	160,216
PBMC10k	scTurboATAC	Seufert et al. [35, 44]	GSE235506	8128	243,114
Hematopoietic cells	scATAC-seq	Satpathy et al. [9, 45]	GSE129785	63,882	571,400
LNCaP	scATAC-seq	Taavitsainen et al. [46, 47]	GSE168667	4436	112,049
K562	SpearATAC	Pierce et al. [48, 49]	GSE168851	32,832	277,112
NeurIPS	Multiome	Luecken et al. [28, 50]	GSE194122	69,249	196,830

The fragment files for the hematopoietic cells dataset [9] were downloaded from GEO with accession number GSE129785. Processed data object with cell barcodes, called peak set, and cell type annotations (scATAC_Heme_All_SummarizedExperiment.final.rds) was downloaded from github (https://github.com/GreenleafLab/10x-scATAC-2019).

The fragment files and processed data objects with cell barcodes, called peak set, and cell type annotations for K562 SpearATAC dataset [48] were downloaded from GEO with accession number GSE168851.

The fragment files for LNCaP dataset [46] were downloaded from GEO with accession number GSE168667.

The fragment files for PBMC10k scTurboATAC dataset [35] were downloaded from GEO with accession number GSE235506.

For the NeurIPS dataset [28], as raw fragment files were not available, the BAM files were downloaded from GEO with accession number GSE194122. Peaks, cell barcodes, and cell type annotations were obtained from the accompanying h5ad file. Sinto (v0.10.1) (https://github.com/timoast/sinto) was used to generate the fragment files.

#### Peak calling and generating PIC matrices for scATAC-seq data

We used the R package “PICsnATAC” v(1.0.0) [6] to generate PIC matrices. The PIC_counting() function requires 3 inputs: (1) fragment file, (2) cell barcodes, and (3) peak set. For hematopoietic cells dataset and K562 SpearATAC dataset, the called peak set and cell barcodes were directly used as input along with the downloaded fragment files. For the NeurIPS dataset, the original peak set was used. For the rest of the datasets, we obtained cell barcodes and peak set by running the default ArchR (v1.0.3) [4] pipeline with the downloaded fragment files as input. For the reference genome, we followed the version that was used to produce the fragment files (Table 3). We filtered cells using default parameters for (minTSS = 4; minFrags = 1000). We then called 500bp peaks using the addReproduciblePeakSet() function with MACS2 as the backend. The resulting cell barcodes and peak set were used as input to generate PIC matrices.

### GC-content normalization

#### GC-content retrieval

We used the Bioconductor R package Biostrings (v2.70.3) [51] to retrieve the GC-content of every peak region, using the reference genome of the relevant dataset. Table 3 provides the genome version used for each dataset.

**Table 3:** Reference genome version used for each scATAC-seq dataset

Dataset	Genome
PBMC10k scATAC-seq	hg38
PBMC10k Multiome	hg38
Hematopoietic cells	hg19
PBMC10k scTurboATAC	hg38
LNCaP	hg38
K562	hg38
NeurIPS	hg38

#### Normalization methods

We adapted code from Van den Berge et al. [5] to test bulk ATAC-seq normalization methods on scATAC-seq data. We tested smooth GC-FQ normalization on both single cell counts and pseudobulked counts. Briefly, smooth GC-FQ is based on full-quantile normalization, which features (1) sorting the counts for each cell, (2) replacing all elements of each feature with its median, and then (3) unsorting each cell. For more details on these methods, please see Van den Berge et al. [5] and Hicks et al. [29].

#### Mock null test

We followed Teo et al. [14], Van den Berge et al. [5] to construct a mock null comparison using the NeurIPS dataset. For each annotated cell type, we randomly split the dataset of 13 donors into 2 groups of 6 and 7 donors each, a mock control and a mock treatment group. Then we used Libra [14] to conduct DA testing between the 2 groups using the Wilcoxon rank sum test.

### Simulation

Our simulation relies on varying the parameters from the hierarchical model in the “A hierarchical model to infer single-cell, single-region chromatin states” section. There are 4 parameters needed to simulate data: (1) observation probability $p_i$, (2) proportion of open cells $\pi _j$, (3) background rate $\lambda ^c_j$, and (4) signal-to-noise ratio $s_j$. In our simulations, we estimated $p_i$ from the hematopoietic cells dataset to represent the sequencing depth variation between cells in real data. $\pi _j$, $\lambda ^c_j$, $s_j$ were either varied as hyperparameters for simulations shown in Fig. 4 or estimated from the PBMC10k multiome dataset for analysis shown in Fig. 5. Below we show how parameters were estimated from data.

#### Varying parameters in silico

For the simulations shown in Fig. 4, we only estimated $p_i$ from the hematopoietic cells dataset (“[Sec Sec27]” section), while varying $\lambda ^c_j$ and $s_j$ in silico. We fixed $\pi _j = 0.3$ for demonstration purposes, but our conclusions hold for other values as well (Additional file 1: Fig. S4). To cover a dynamic range of parameter values, we simulated data with $\lambda ^c_j \in \{0.001,0.005,0.01, 0.015, 0.02\}$ and $s_j \in \{2,5,10,50,100\}$. For each combination of $\lambda ^c_j$ and $s_j$, the simulation was repeated for 30 times and the mean AUROC is reported (“Computation of posterior probability” section).

#### Estimating observation probability $p_i$

Observation probability $p_i$ was estimated from the hematopoietic cells dataset using the PIC model [6]. Below we adapt notations from Miao and Kim [6] to stay consistent with our previous definitions. Briefly, the PIC model introduces a binary vector $\mathbf {T_i}$ that indicates whether a genomic region *j* is measured in a cell *i*. Whether a region is measured depends on the observation probability $q_i$ (Eq. 8),

8 $$\begin{aligned} \mathbf {T_i} \sim \text {Bernoulli}(q_i) . \end{aligned}$$

Although conceptually similar to our binomial measurement model (Eq. 5), the PIC measurement model assumes an “all-or-nothing” mechanism—Tn5 insertion events are either all observed or all dropped out. Realistically, the more underlying insertion events there are in a region, the less likely all events in that region are dropped out. However, inference for $p_i$ in Eq. 5 has no closed form solution and for data with generally low counts, $q_i$ should be a good approximation for $p_i$. Therefore, we used the get_r_by_ct_mat_pq() function from the “PICsnATAC” R package to estimate $q_i$, and used the estimated $q_i$ as our observation probability $p_i$. For the simulations shown in Fig. 4, we randomly sampled 10,000 observations from the estimated $p_i$ to simulate from.

#### Estimating background rate $\lambda ^c_j$

Background regions were used to infer $\lambda _j^c$. We chose background regions by using regions 500bp upstream and downstream of called peaks. Let $k \in \{1, \dots , M\}$ index background regions. We assumed the same data generative process as our main model but all cells are closed in these regions, i.e., $\pi _k=0$, such that all counts are due to background rate $\lambda ^c_k$. Then $\lambda ^c_k$ can be solved by matching the first moment. We denote $\bar{x}_k$ as the empirical mean of background region *k* and $\bar{p}$ as the empirical mean of the previously estimated $p_i$:

$$\begin{aligned} \bar{x}_k= &\ \mathbb {E}[x_{ik}] \\ \bar{x}_k= &\ \mathbb {E}[y_{ik}p_{i}]\\ \bar{x}_k= &\ \mathbb {E}[y_{ik}]\mathbb {E}[p_{i}]\\ \bar{x}_k= &\ \lambda ^c_k\bar{p} \\ \lambda ^c_k= &\ \frac{\bar{x}_k}{\bar{p}} . \end{aligned}$$

To model the effect of GC-content on background rate, we fit the following generalized additive model (GAM) using GC-content of each background region ($\text {GC}_k$) as the predictor variable using “mgcv” R package:

9 $$\begin{aligned} \lambda ^c_k = \beta _0 + f(\text {GC}_k) + \epsilon _k \quad \text {where} \epsilon _k \sim N(0, \sigma ^2). \end{aligned}$$

To prevent the fit from being affected by background regions with extremely high counts, background regions with $\lambda ^c_k$ larger than 10 times the interquartile range were not used to fit the GAM. A total of 67,433 background regions (5.9% of all background regions) were filtered because of this reason. Lastly, to obtain an estimate for $\lambda ^c_j$, we use the fitted GAM to predict $\hat{\lambda }^c_j$ using the GC-content of called peaks ($\text {GC}_j$).

#### Estimating proportion of open cells $\pi _j$ and signal-to-noise ratio $s_j$

Estimating $\pi _j$ and $s_j$ from data is tricky as $p_i$ is a cell-specific parameter, which makes each cell-peak pair a unique function of $\pi _j$, $s_j$ and $p_i$ with 1 single observation $x_{ij}$. In this case, $\pi _j$ and $s_j$ are unidentifiable.

However, they are identifiable if $p_i$ is a constant instead. If we pool cells together with a similar $p_i$ and replace it with the empirical mean $\bar{p}$, then $\pi _j$ and $s_j$ for that pool can be solved. We pooled the data into bins of 100 cells and estimated parameters for each pool separately, then took the mean of the estimates to obtain the final estimate. Briefly, for each pool *w*,

10 $$\begin{aligned} p^w = \bar{p_i^w}, \end{aligned}$$

11 $$\begin{aligned} \hat{\pi ^w_j}, \hat{s^w_j} = \text {argmin}(\Vert p(x|\pi ^w_j, s^w_j) - \hat{p}(x) \Vert _2) . \end{aligned}$$

After obtaining estimates for each pool, the final estimate can be obtained by:

12 $$\begin{aligned} \hat{\pi _j} = \bar{\hat{\pi ^{w}_j}}, \end{aligned}$$

13 $$\begin{aligned} \hat{s_j} = \bar{\hat{s^w_j}} . \end{aligned}$$

#### Computation of posterior probability

The posterior probability of cell *i* being “open” in region *j* is given by:

$$\begin{aligned} P(Z_{ij} = 1 \mid x_{ij}, \pi _{j}, \lambda ^c_{j}, s_j, p_{i}) = \frac{\pi _j p(x_{ij}| \lambda ^c_{j}, s_j, p_{i})}{\pi _j p(x_{ij}| \lambda ^c_{j}, s_j, p_{i}) + (1-\pi _j)p(x_{ij} | \lambda ^c_{j}, p_{i})} . \end{aligned}$$

The marginal p.d.f of $x_{ij}$ is given by:

$$\begin{aligned} p(x_{ij} = k | \lambda ^c_{j}, s_j, p_{i})= &\ \sum \limits ^{\infty }_{k^{\prime }=k} P(y_{ij} = k^{\prime }) P(x_{ij} = k \mid y_{ij} = k^{\prime })\\= &\ p_{i}^k \sum \limits ^{\infty }_{k^{\prime }=k} \frac{(\lambda ^{c}_{j}s_{j})^{k^{\prime }}e^{-\lambda }}{k^{\prime }!} \left( {\begin{array}{c}k^{\prime }\\ k\end{array}}\right) (1-p_{i})^{k^{\prime }-k} . \end{aligned}$$

In a realistic scenario, the parameters should be estimated from data to calculate the posterior. However, in our simulations, we calculated the posterior with ground truth parameters (i.e., assuming perfect recovery of parameters). Note that the integral for the marginal p.d.f has no closed form solution. Therefore, for computational purposes it is approximated by addition up to 50. Further note that for the marginal p.d.f of the closed component, $s_j = 1$.

#### Computation of AUROC

The AUROC is calculated using the “pROC” (v1.18.5) [52] package in R with default parameters. We used simulated chromatin states of each cell as response and the posterior probability as the predictor.

### PBMC10k multiome analysis

We first performed brief quality control on the data, by filtering cells with total ATAC count $\ge$ 100,000, total RNA count $\ge$ 25,000 and total RNA count $\le$ 1000. Then we removed doublets using scDblFinder (v1.20.2) [53] with default settings. Peak counts are aggregated according to their annotated nearest gene, resulting in a gene $\times$ cell count matrix. Then the posterior probability for each gene is calculated as described above. PCA was done using prcomp() function in R on the posterior probability matrix. LSI in Fig. 5 is performed using the Signac (v1.13.0) [8] pipeline according to the Signac vignette. Briefly, peaks with more than 5 counts are subject to TF-IDF normalization, then Singular Value Decomposition (SVD) is performed to reduce the dimensions of the data. To annotate the multiome data, we followed the same vignette and transferred labels from an annotated atlas [54]. Silhouette score is calculated with the approxSilhouette() function in the “bluster” R package (v1.16.0) [55], with the first 30 principal components as input for both posterior and LSI processed data. The mean silhouette for each cell type is shown in each boxplot in Fig. 5. Silhouette width for each cell, for each cell type, is shown in Additional file 1: Fig. S12.

## Supplementary Information

Additional file 1. Supplementary figures.

## Data Availability

Data and code to reproduce the figures in this manuscript are available (under CC BY 4.0 license) at the following Gitlab repository: https://gitlab.svi.edu.au/biocellgen-public/gath_2023_scatac_mixture_modelling_reproducibility [56] and Zenodo (https://doi.org/10.5281/zenodo.15876051) [57]. Accession to third-party datasets and publicly available datasets used is listed in Table 2.
