# Supplementary material for: A hierarchical, count-based model highlights challenges in scATAC-seq data analysis and points to opportunities to extract finer-resolution information
Source: Genome Biol. 2025 Sep 17;26:282. doi: 10.1186/s13059-025-03735-y (PMC12442292; doi:10.1186/s13059-025-03735-y)
Supplement: Supplementary file 1 — Additional file 1. Supplementary figures. [file 13059_2025_3735_MOESM1_ESM.pdf]

Supplementary figures

July 2025

List of Figures

S1 Histogram of paired-insertion counts (PIC) for all datasets analysed. 1
S2 Log fold change against GC bins for all cell types in NeurIPS dataset. 2
S3 Log fold change against GC bins for CD16+ Monocytes in NeurIPS dataset. Shown here is the result for 20 random splits of donors. 3
S4 Simulations from Fig. 4 with varying π. The performance of the model (as measured by the mean AUROC) is mostly invariant to π, the proportion of open cells. 4
S5 Ratio of probabilities of sampling a 0 from closed cells vs open cells i.e. $P(X = 0|Z_{ij} = 0)/P(X = 0|Z_{ij} = 1)$, with varying background rates and signal-to-noise ratios. As background rate increases, the likelihood of sampling a 0 from a closed cell is exponentially higher than a open cell. 5
S6 Pairs plot visualisation of PCs derived from model posterior coloured by cell types. First 5 PCs are shown here. 6
S7 Pairs plot visualisation of LSI components coloured by cell types. First 5 components are shown here. 7
S8 UMAP visualisation of data processed with LSI and model posterior, coloured by cell types. 8
S9 Scree plot showing percentage of variance explained for LSI and posterior. 8
S10 Visualisation of first 2 PCs from LSI and model posterior, coloured by log LS. 9
S11 UMAP visualisation of LSI and model posterior, coloured by log10 library size. 10
S12 Silhouette width for each cell type for each method. 11
S13 Log10 library size for each cell type. 12
S14 Silhouette widths against log10 library size for each cell type using the posterior approach. 13

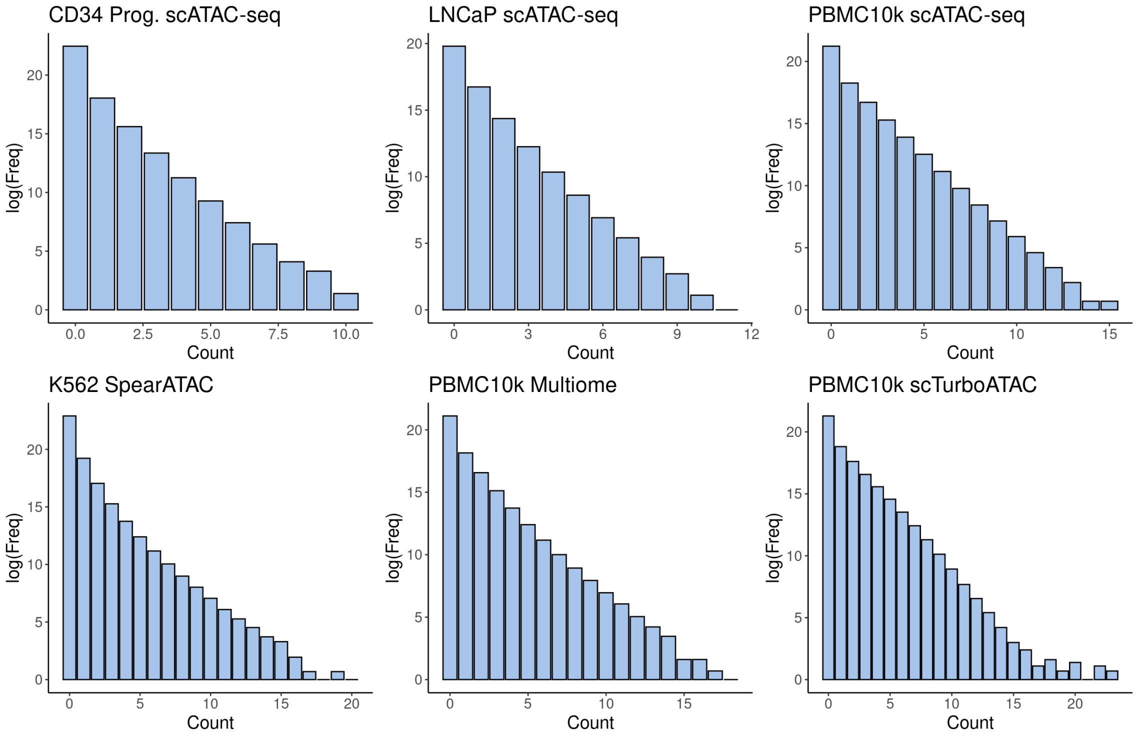

Figure S1: Histogram of paired-insertion counts (PIC) for all datasets analysed.

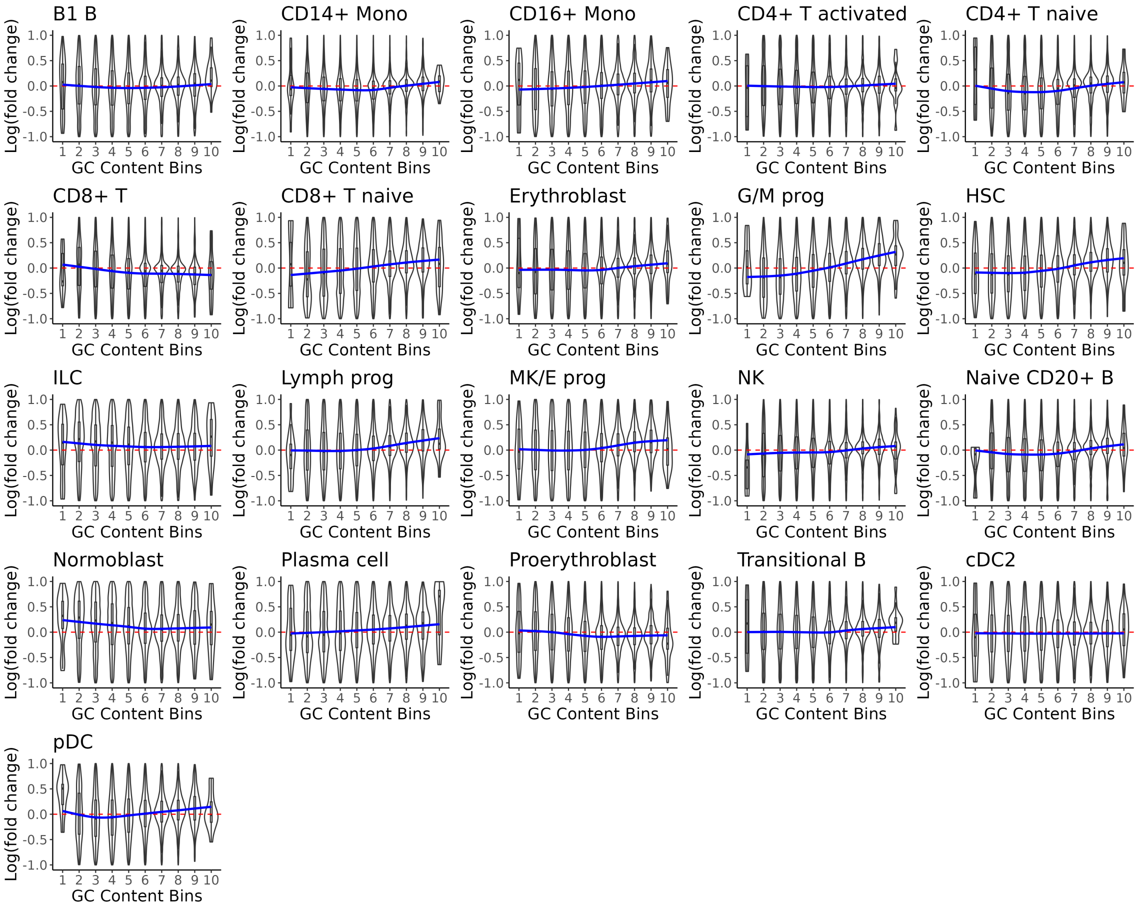

Figure S2: Log fold change against GC bins for all cell types in NeurIPS dataset.

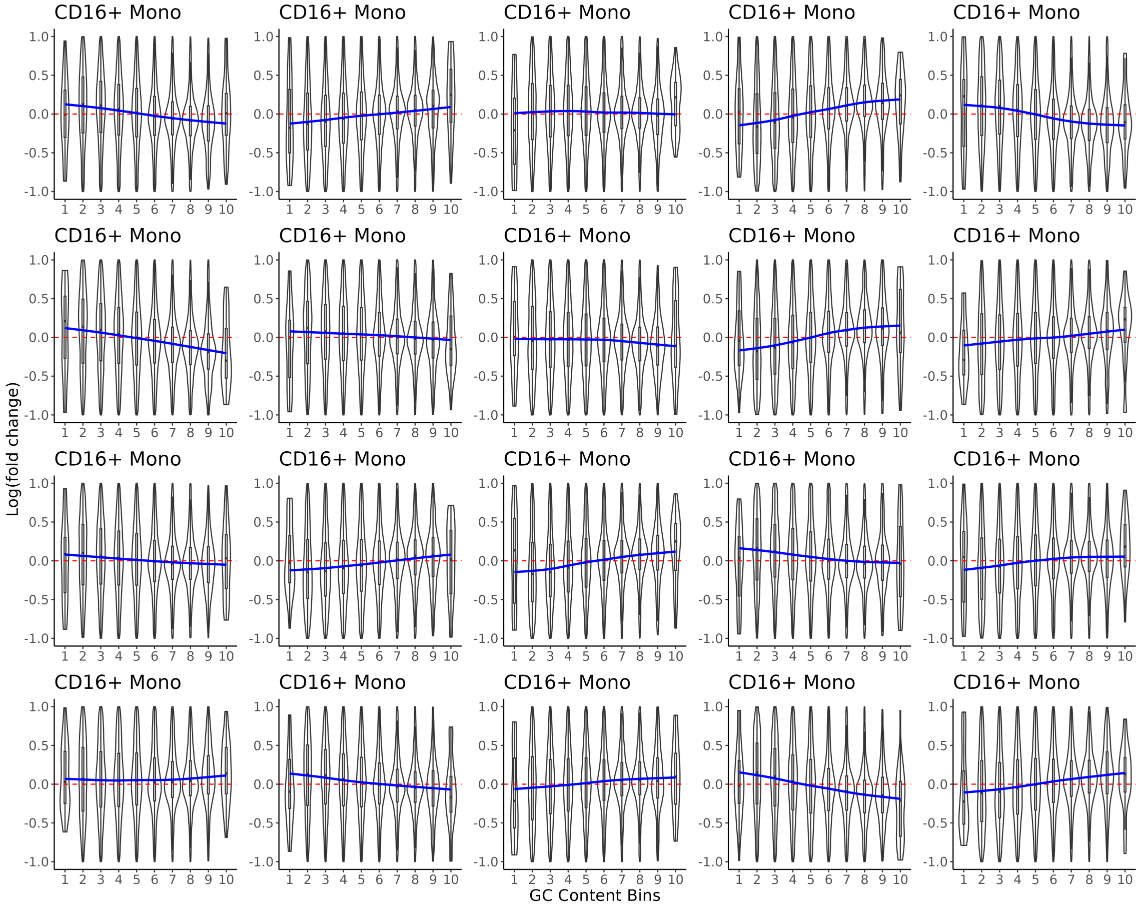

Figure S3: Log fold change against GC bins for CD16+ Monocytes in NeurIPS dataset. Shown here is the result for 20 random splits of donors.

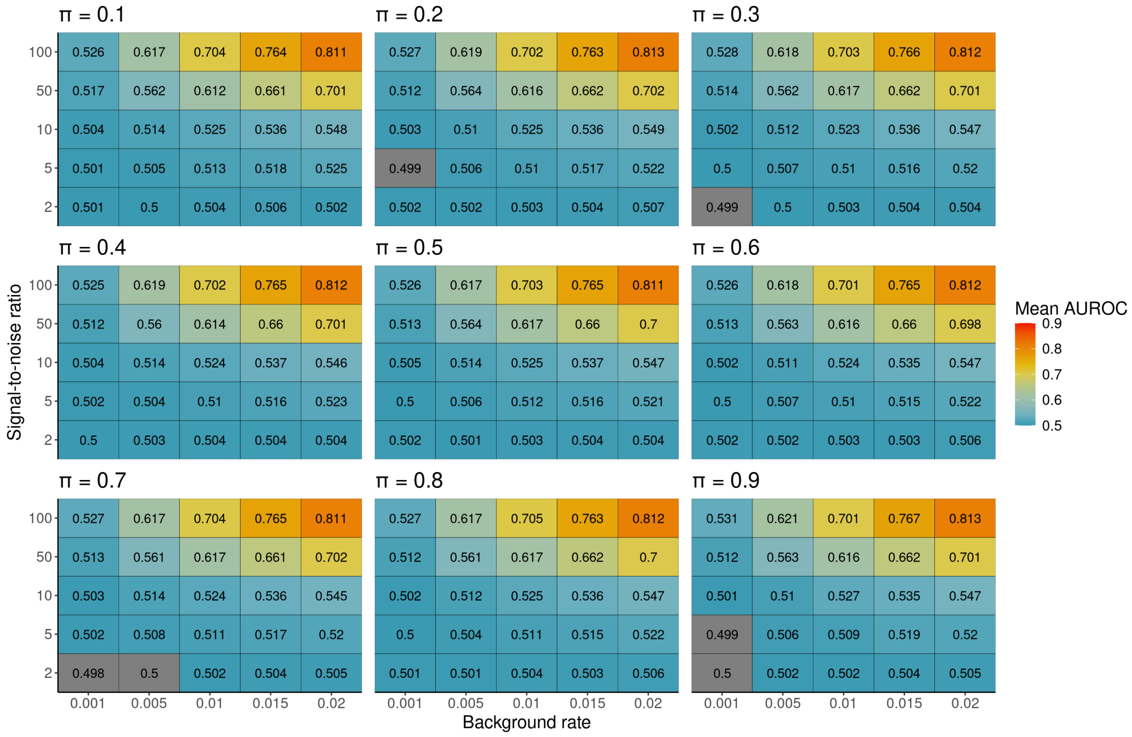

Figure S4: Simulations from Fig. 4 with varying π. The performance of the model (as measured by the mean AUROC) is mostly invariant to π, the proportion of open cells.

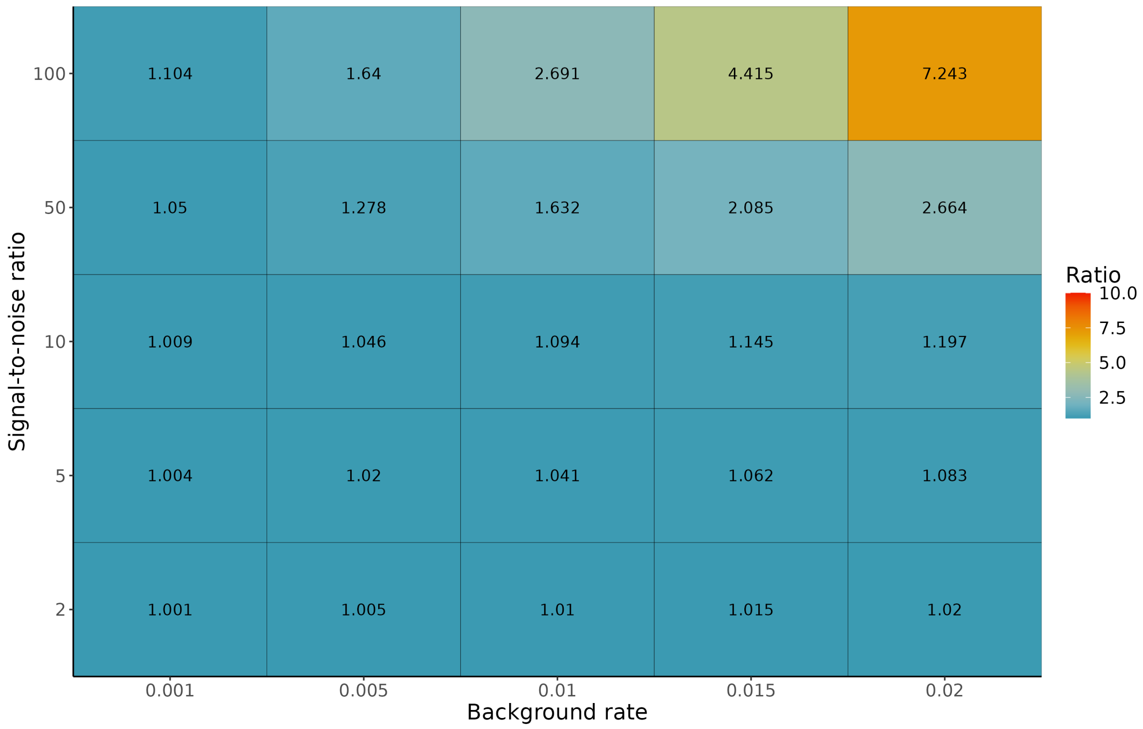

Figure S5: Ratio of probabilities of sampling a 0 from closed cells vs open cells i.e. $P(X = 0|Z_{ij} = 0)/P(X = 0|Z_{ij} = 1)$, with varying background rates and signal-to-noise ratios. As background rate increases, the likelihood of sampling a 0 from a closed cell is exponentially higher than a open cell.

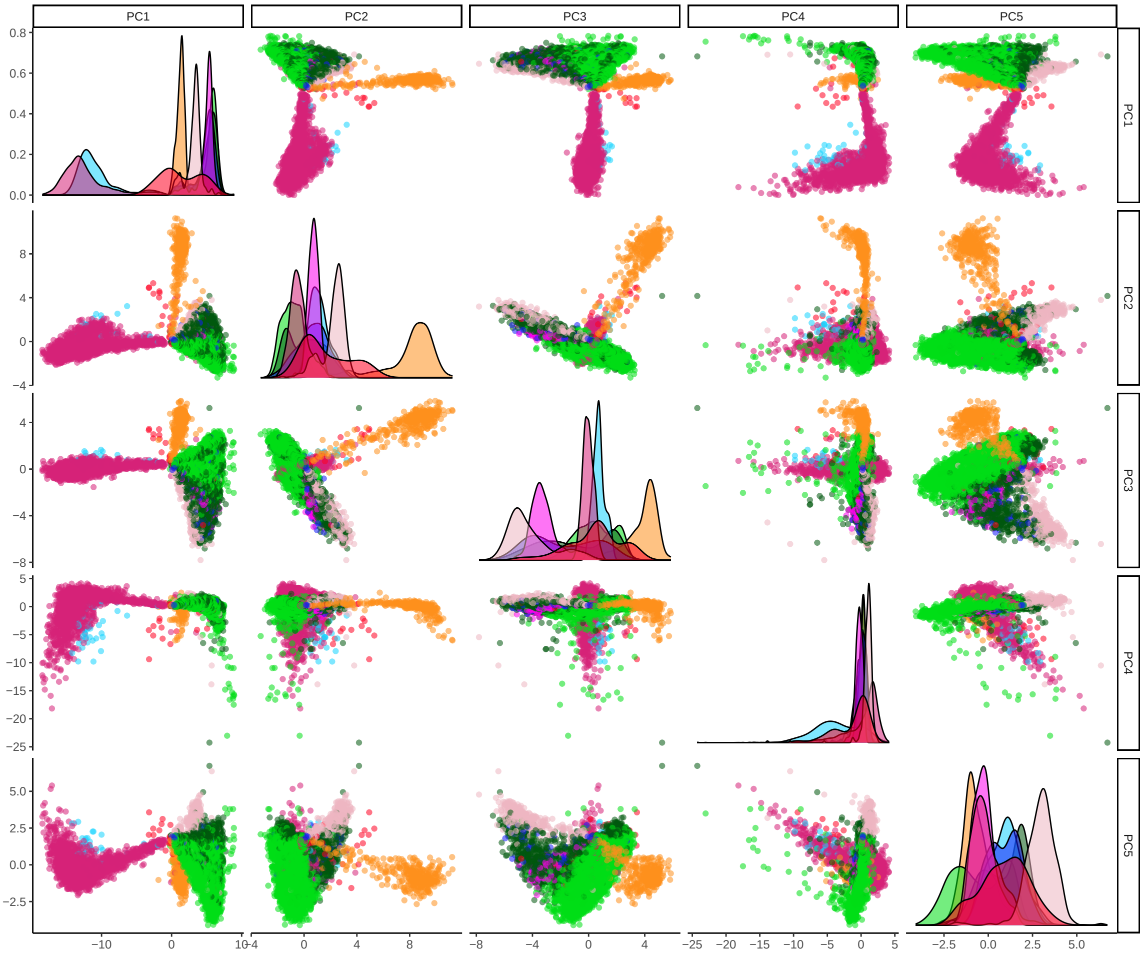

Figure S6: Pairs plot visualisation of PCs derived from model posterior coloured by cell types. First 5 PCs are shown here.

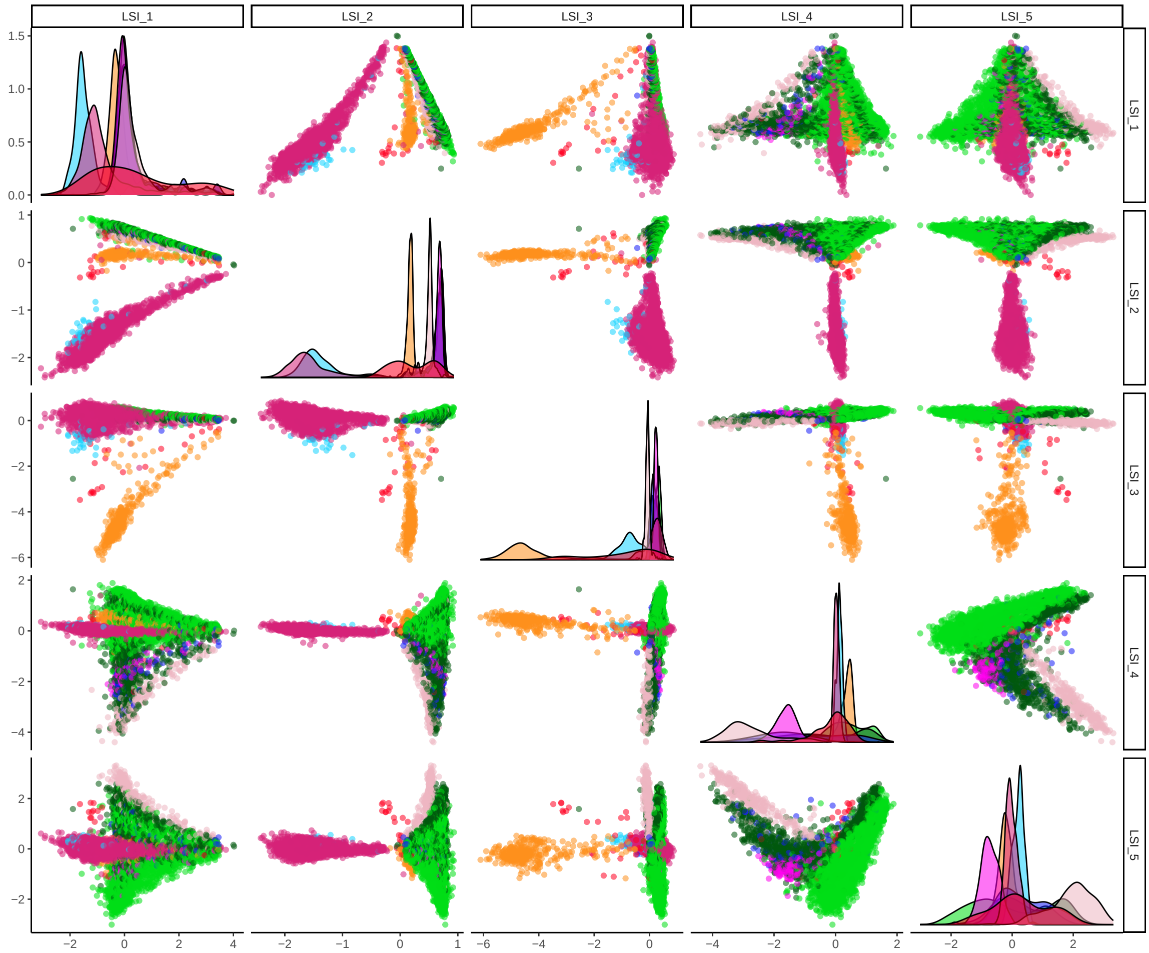

Figure S7: Pairs plot visualisation of LSI components coloured by cell types. First 5 components are shown here.

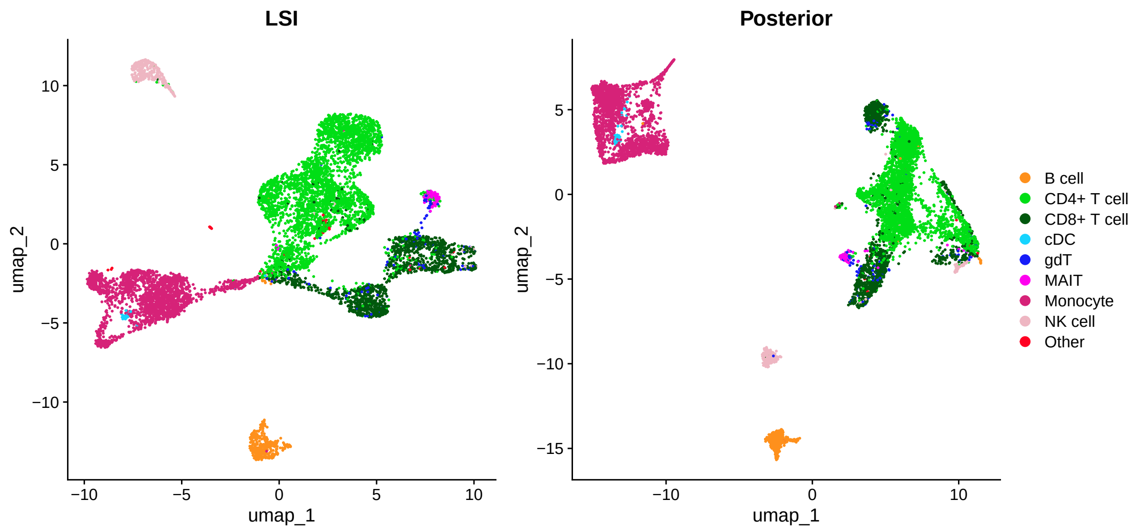

Figure S8: UMAP visualisation of data processed with LSI and model posterior, coloured by cell types.

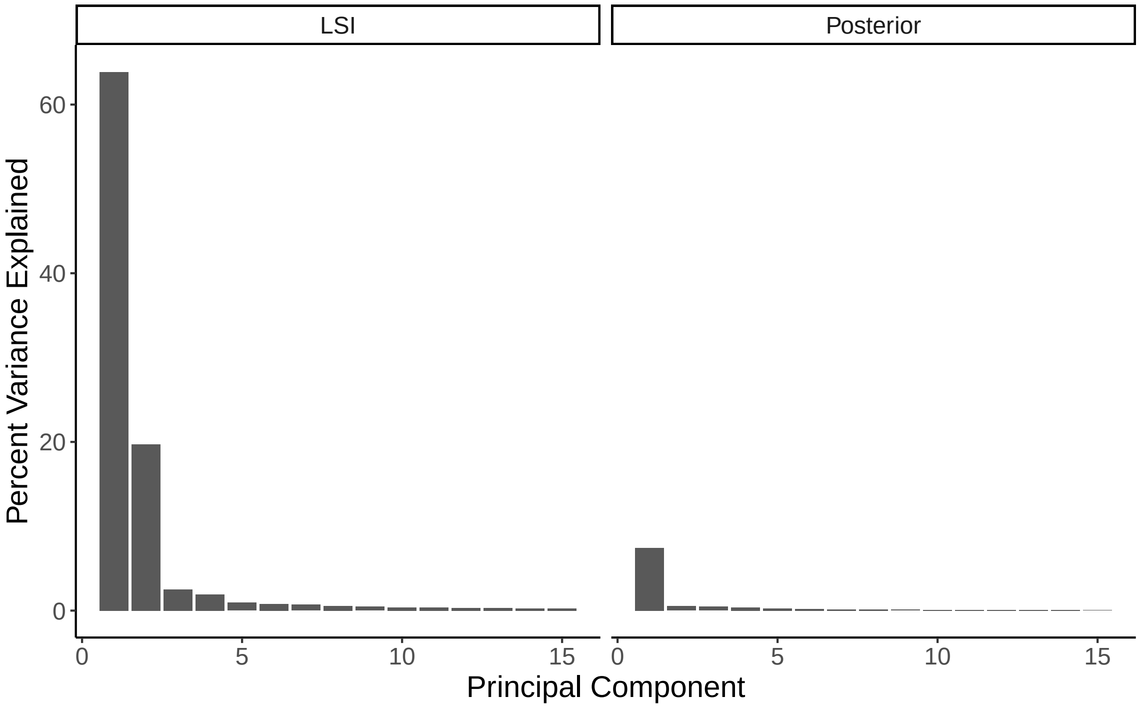

Figure S9: Scree plot showing percentage of variance explained for LSI and posterior.

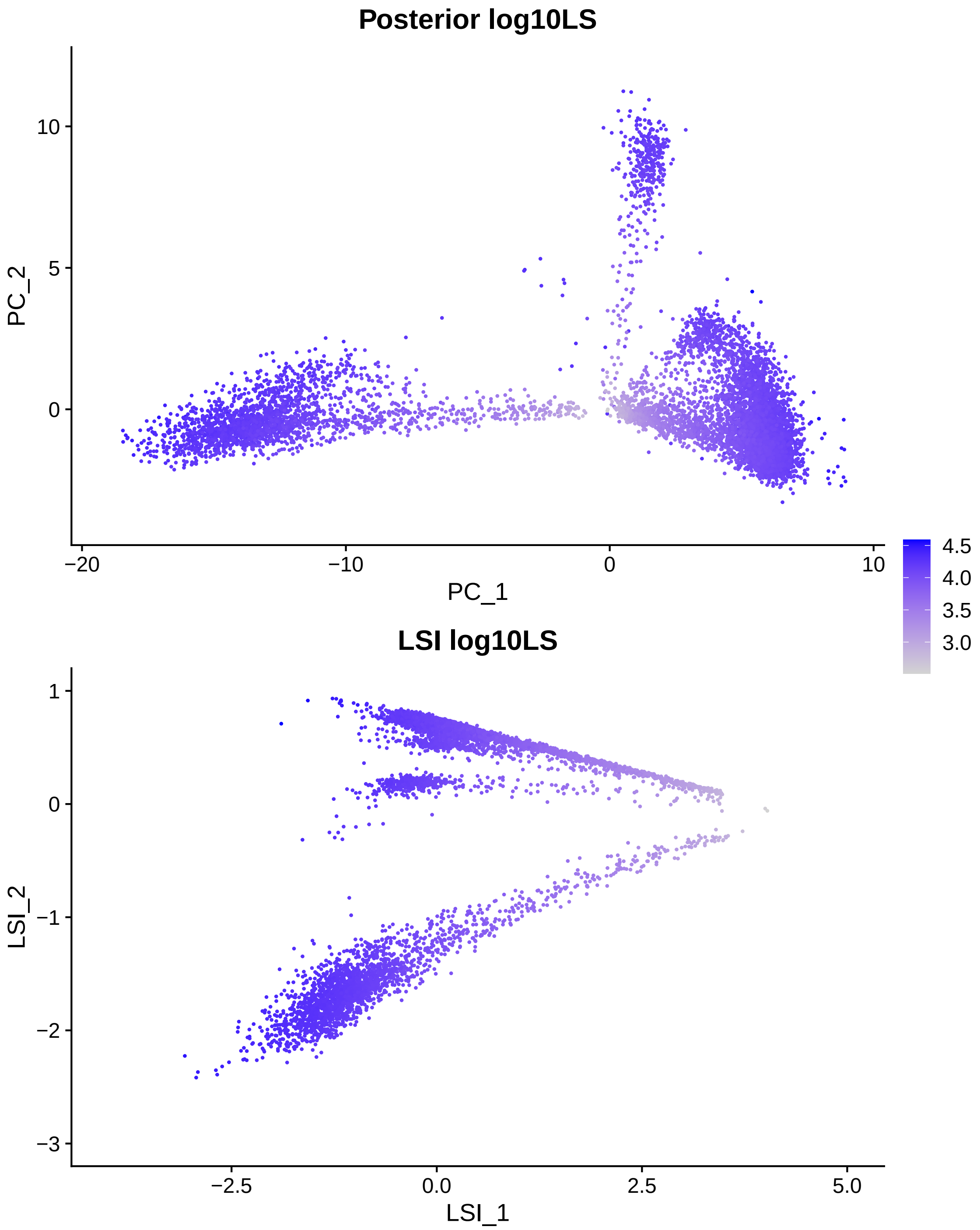

Figure S10: Visualisation of first 2 PCs from LSI and model posterior, coloured by log LS.

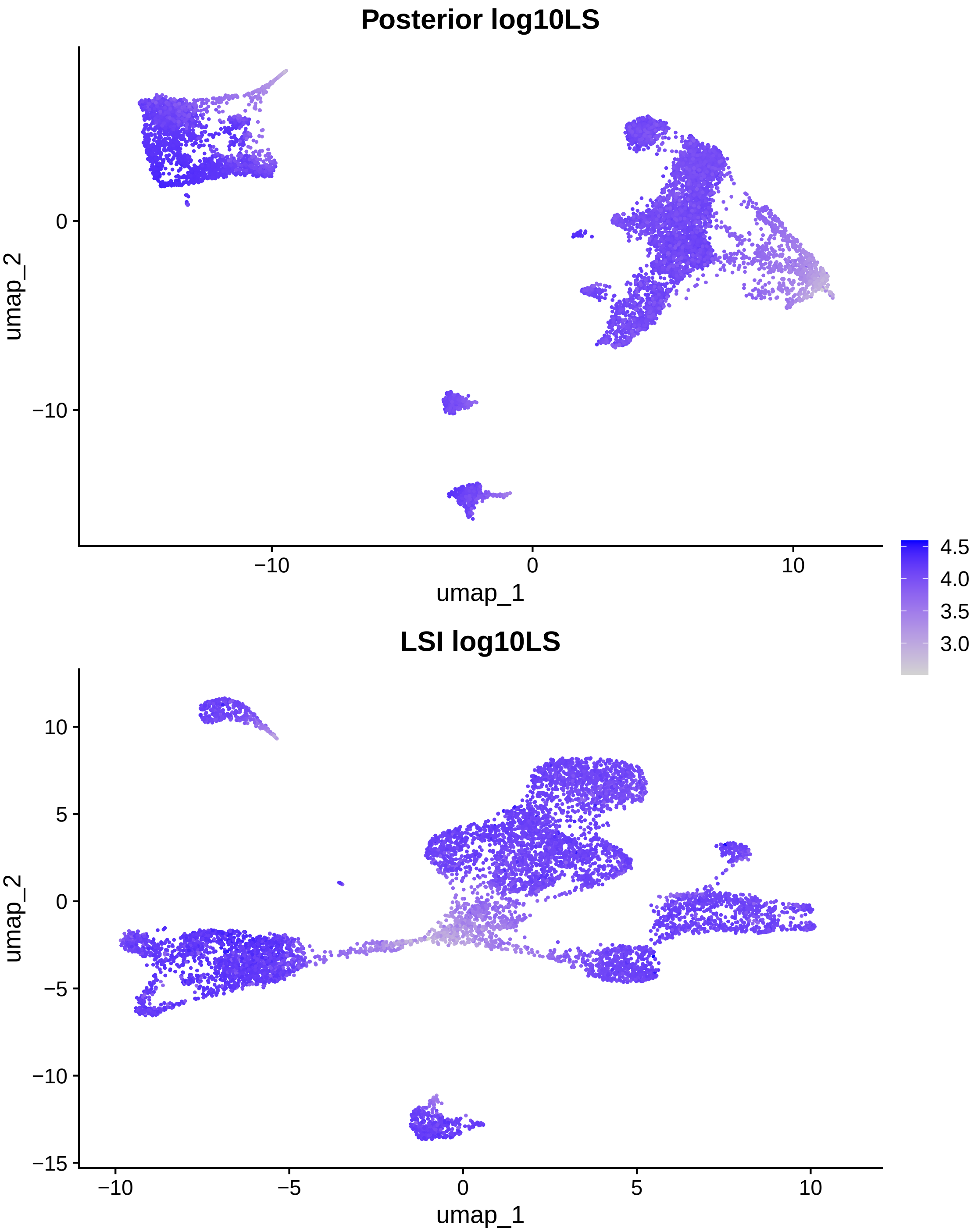

Figure S11: UMAP visualisation of LSI and model posterior, coloured by log10 library size.

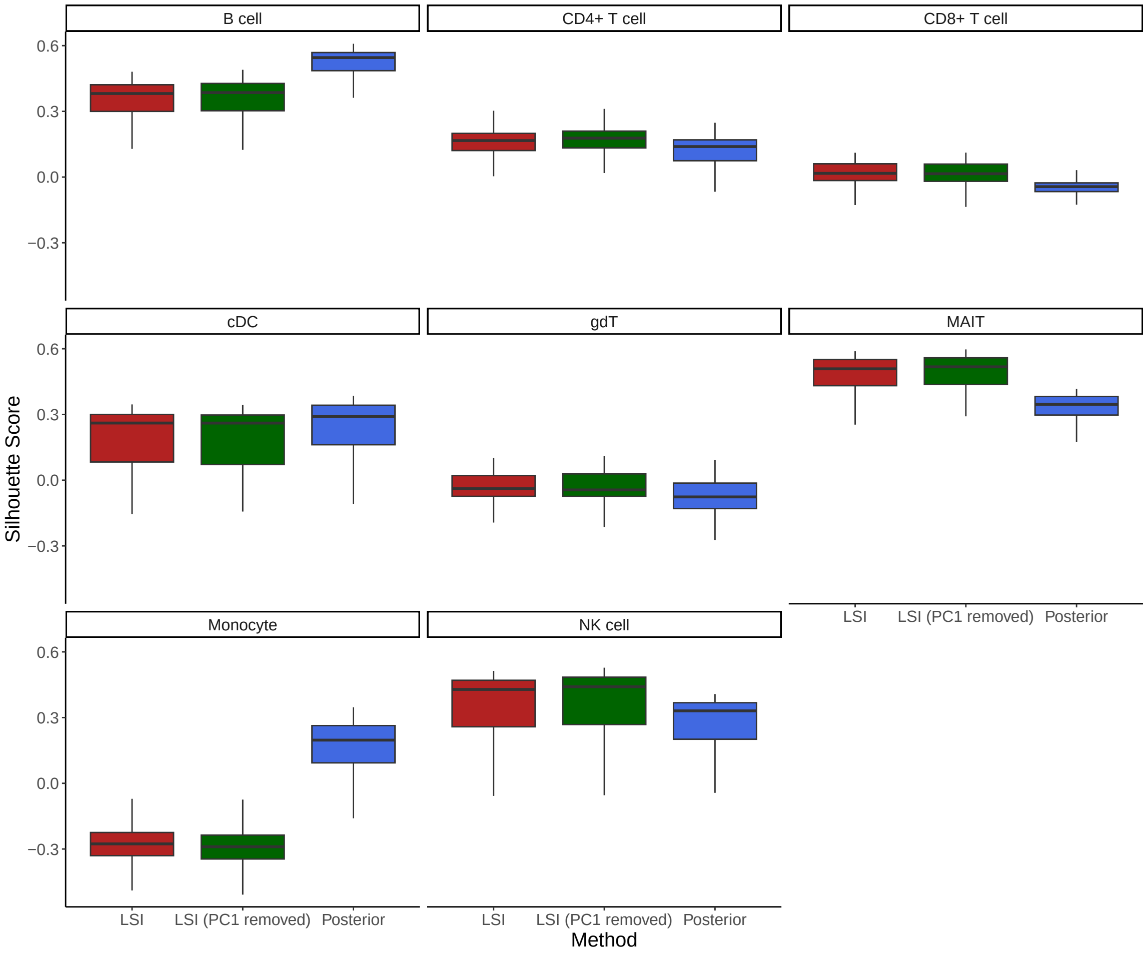

Figure S12: Silhouette width for each cell type for each method.

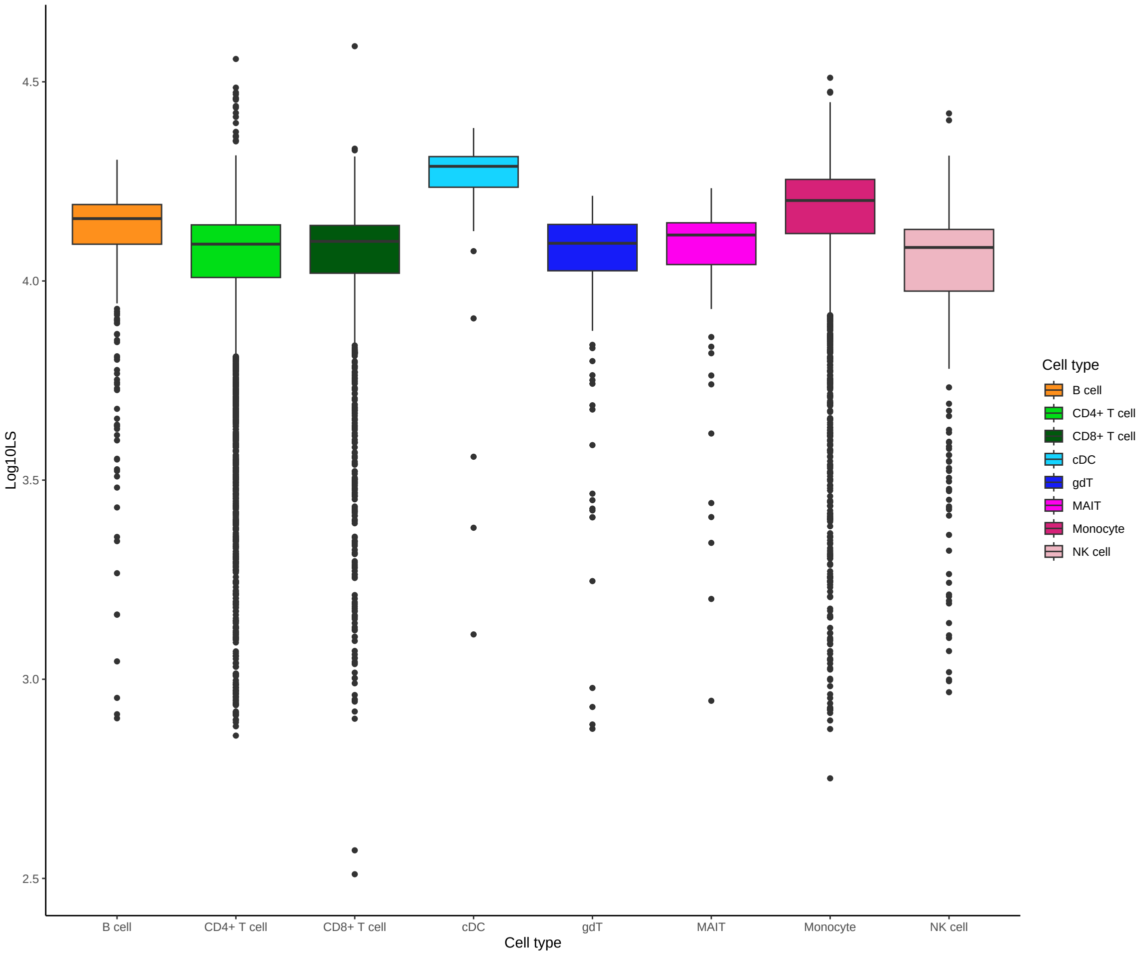

Figure S13: Log10 library size for each cell type.

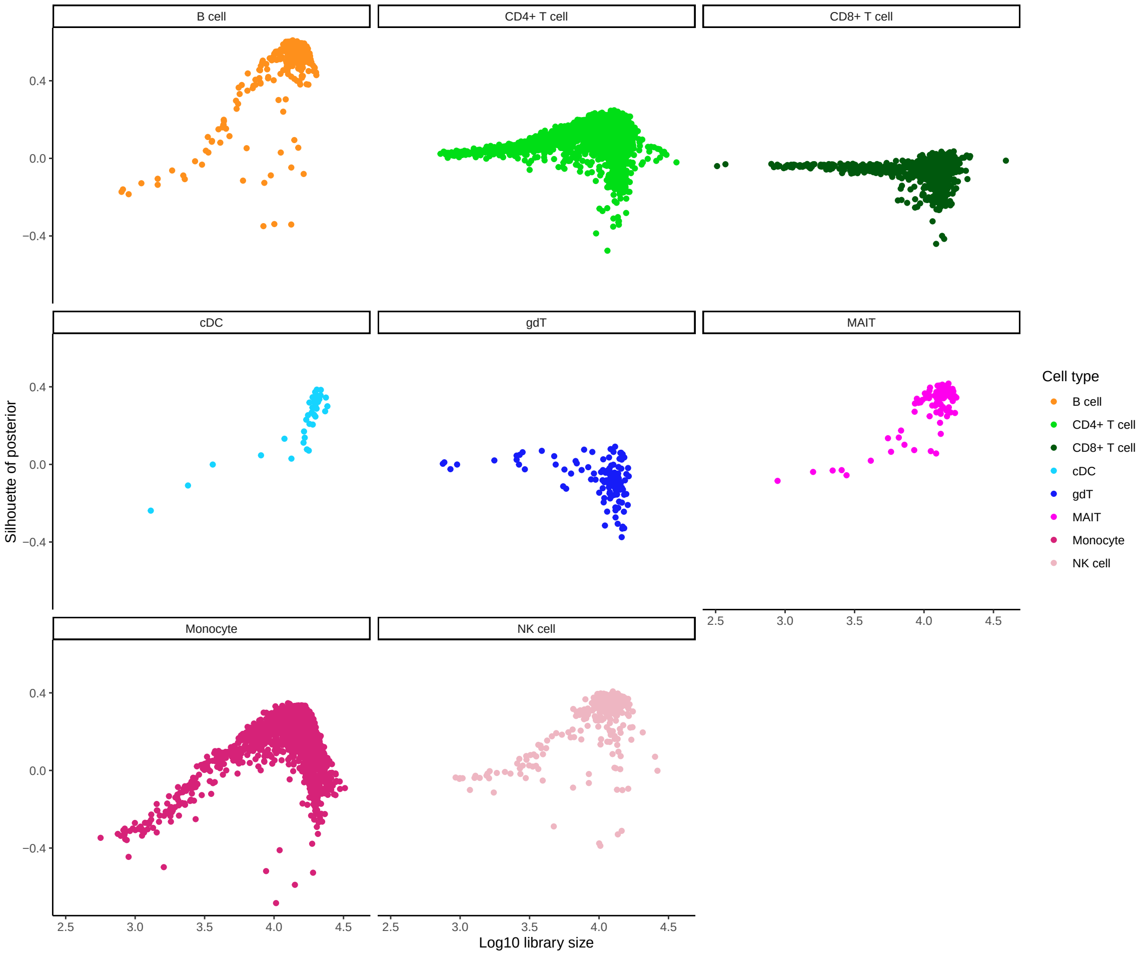

Figure S14: Silhouette widths against log10 library size for each cell type using the posterior approach.
